# Integrating topic modeling and word embedding to characterize violent deaths

**DOI:** 10.1073/pnas.2108801119

**Published:** 2022-03-03

**Authors:** Alina Arseniev-Koehler, Susan D. Cochran, Vickie M. Mays, Kai-Wei Chang, Jacob G. Foster

**Affiliations:** ^a^Department of Sociology, University of California, Los Angeles, CA 90095;; ^b^Bridging Research Innovation, Training and Education for Science, Research & Policy Center, University of California, Los Angeles, CA 90095;; ^c^Department of Epidemiology, Fielding School of Public Health, University of California, Los Angeles, CA 90095;; ^d^Department of Statistics, University of California, Los Angeles, CA 90095;; ^e^Department of Psychology, University of California, Los Angeles, CA 90095;; ^f^Department of Health Policy and Management, Fielding School of Public Health, University of California, Los Angeles, CA 90095;; ^g^Department of Computer Science, University of California, Los Angeles, CA 90095

**Keywords:** natural language processing, mortality surveillance, gender, topic models, word embeddings

## Abstract

There is an escalating need for methods to identify latent patterns in text data from many domains. We introduce a method to identify topics in a corpus and represent documents as topic sequences. Discourse atom topic modeling (DATM) draws on advances in theoretical machine learning to integrate topic modeling and word embedding, capitalizing on their distinct capabilities. We first identify a set of vectors (“discourse atoms”) that provide a sparse representation of an embedding space. Discourse atoms can be interpreted as latent topics; through a generative model, atoms map onto distributions over words. We can also infer the topic that generated a sequence of words. We illustrate our method with a prominent example of underutilized text: the US National Violent Death Reporting System (NVDRS). The NVDRS summarizes violent death incidents with structured variables and unstructured narratives. We identify 225 latent topics in the narratives (e.g., preparation for death and physical aggression); many of these topics are not captured by existing structured variables. Motivated by known patterns in suicide and homicide by gender and recent research on gender biases in semantic space, we identify the gender bias of our topics (e.g., a topic about pain medication is feminine). We then compare the gender bias of topics to their prevalence in narratives of female versus male victims. Results provide a detailed quantitative picture of reporting about lethal violence and its gendered nature. Our method offers a flexible and broadly applicable approach to model topics in text data.

## Significance

We introduce an approach to identify latent topics in large-scale text data. Our approach integrates two prominent methods of computational text analysis: topic modeling and word embedding. We apply our approach to written narratives of violent death (e.g., suicides and homicides) in the National Violent Death Reporting System (NVDRS). Many of our topics reveal aspects of violent death not captured in existing classification schemes. We also extract gender bias in the topics themselves (e.g., a topic about long guns is particularly masculine). Our findings suggest new lines of research that could contribute to reducing suicides or homicides. Our methods are broadly applicable to text data and can unlock similar information in other administrative databases.

Digital technologies have produced a deluge of computer-readable text: tweets, blogs, legal documents, product reviews, scientific articles, financial reports, electronic health records, and administrative records (e.g., from public health surveillance). Despite its promise, deriving meaningful information from large-scale text remains a challenge (1). This is especially so in real-world applications, which often put particular demands on methods for computational text analysis. Such methods should be interpretable. They should adapt to the nuances of different discourses. And they should have strong theoretical foundations. In this paper, we offer an approach that meets these demands: discourse atom topic modeling (DATM). DATM integrates topic modeling (2) and word embedding (3) to identify latent topics in embeddings and map documents onto topics. Methods developed for embeddings (e.g., latent dimensions of cultural meaning) (4, 5) can be applied directly to the topics. We illustrate the value of DATM using text data collected through an ongoing public-health surveillance system for lethal violence in the United States.

Violent death surveillance provides a striking example of the promise and challenge of computational text analysis. Violent deaths are among the leading causes of mortality in the United States (6): More than seven people per hour die a violent death (7). Understanding and reducing the frequency of these deaths are major goals for public health. Much of what we know about violent death comes from large administrative databases like the National Violent Death Reporting System (NVDRS), a nationwide public-health surveillance dataset established by the Centers for Disease Control (CDC) in 2003 (8, 9). The NVDRS contains both structured variables (e.g., victim demographics) and unstructured text narratives. These narratives describe the circumstances of death incidents based on reports from law enforcement, medical examiners/coroners, toxicology, and crime laboratories. While much has been learned from the NVDRS, researchers have largely used the structured variables; traditional qualitative methods are too labor intensive to use at scale. The narratives, summarizing more than 300,000 violent deaths, remain mostly unused, despite their potential to illuminate many aspects of violent death, from proximate correlates to nuanced context.

Consider a well-known and durable pattern: differences in violence by gender. Men are more likely than women to die from and perpetrate lethal violence (10, 11). Men and women victims also tend to die by different methods (11, 12). Among suicides and homicides, for example, men are more likely to use firearms, while women are more likely to use poisonous substances (11, 12). While such gender-linked patterns are reflected across structured variables in the NVDRS (and are well documented in the literature), the NVDRS narratives may also encode gendered patterns—some as yet unidentified. Gendered patterns in text are expected; a growing body of computational work illustrates how and how often information about gender manifests in language (4, 5).

The case of violent death surveillance highlights two problems that computational text analysis can solve. First, researchers often want to summarize large corpora, e.g., by extracting major themes like “hot” scientific topics in PNAS (13). Second, researchers want to find evidence for patterns suggested by theory or prior scholarship, e.g., the presence and dynamics of gender and ethnic stereotypes in media language (4). Existing methods can solve both of these problems, but separately. DATM enables us to do both at once. It integrates two major innovations in computational text analysis: topic modeling (2) and word embedding (3).

Topic modeling methods identify latent themes in a corpus and connect those themes to observed words and documents. In conventional topic modeling, topics are distributions over words, and documents are distributions over topics. Powerful as they are, existing topic modeling approaches—especially those commonly used in computational social science—remain largely disconnected from contemporary strategies to represent semantic information using word embeddings. For details and exceptions, see *SI Appendix*.

Word-embedding methods represent word meanings by mapping each word in the vocabulary to a point in an *N*-dimensional semantic space (a “word vector”). Words used in similar contexts in the corpus are mapped to nearby points. In a well-trained embedding, word vectors represent semantic information in ways that correspond to human meanings. For example, words that humans rate as similar tend to be closer in semantic space. While word embeddings explicitly model words, they also encode latent semantic structures, like dimensions that correspond to gendered meanings (4, 5, 14); analysts can use these dimensions to quantify the latent meanings (e.g., gender) of all the words in a corpus. Topic modeling and word embedding thus have distinct strengths and limitations.

DATM identifies topics (latent themes) and infers the distribution of topics in a specific document, just like a standard topic model. Unlike standard topic modeling, however, DATM does so in an explicit embedding framework; both words and topics live in one semantic space. Our method therefore offers rich representations of topics, words, phrases, and latent semantic dimensions in language. It does so by integrating several theoretical advances to explain word embeddings and efficiently represent sentences in semantic space (15–18).

After describing DATM, we use it to identify key topics in narratives describing over 300,000 violent deaths in the NVDRS (2003 to 2017). We observe a range of topics, including ones about family, preparation for death, and causality. Using recent approaches to identify semantic dimensions in embedding space (5, 14), we identify a gender dimension and compute the gendered meanings of our topics. We describe two illustrative topics in depth: 1) rifles and shotguns and 2) sedative and pain medications. Our approach allows us to summarize and contextualize large-scale, unstructured accounts of violent death. It also allows us to zoom in on “needles” in this haystack of data (19) and investigate patterns suggested by theory or prior scholarship.

## Integrating Topic Modeling and Word Embedding

To integrate topic modeling and word embedding, we address two core methodological challenges. First, we identify latent topics in a trained word embedding space (also referred to as semantic space); here, we set out to identify topics in an embedding space trained on narratives of violent death. Second, we identify the topic(s) underlying an observed set of words (e.g., a sentence, document, or death narrative). More generally, we need a theoretical framework to connect an embedding space to raw text data. DATM integrates several methodological and theoretical advances in research on word embeddings to address these two challenges, as described next.

### Identifying Topics in Semantic Space.

We begin with a word embedding trained on a specific corpus (in our case, narratives of violent death). To identify topics in this embedding space, we apply K-singular value decomposition (K-SVD), a sparse dictionary learning algorithm (20, 21), to the word vectors (16). This algorithm outputs a set of *K* vectors (called “discourse atoms” by ref. 16) such that any of the *V* word vectors in the vocabulary can be written as a sparse linear combination of atom vectors. Using the generative model below (Eq. **1**), atom vectors can be interpreted as topics in the embedding space. The words closest to each atom vector characterize the topic.

We apply K-SVD to our word embedding while varying the number of atom vectors *K*. To select a final sparse representation, we use a combination of previously proposed metrics for topic model quality and an additional metric suitable for K-SVD (*R*^2^). Together, these metrics quantify 1) how internally coherent topics are, 2) how distinct topics are from each other, and 3) how well the underlying atoms explain the semantic space itself. We select our final model (with 225 topics) to balance performance across these metrics. See *SI Appendix* for details and for a comparison with other topic-modeling approaches.

### Moving from Semantic Space to Text Data and Back.

Sparse dictionary learning offers a way to identify the “building blocks” of semantic space, but it does not map observed sequences of words (e.g., sentences) to these building blocks. Fortunately, a recently proposed language model offers a link between observed words and points in semantic space: the latent variable model (15, 17). This model provides a simplified, probabilistic account for how the text in a corpus was generated. But it also provides a theoretically motivated algorithm to summarize a given set of words as a context vector in the semantic space, i.e., a sentence embedding (17, 18). For a given context vector, we can find the closest atom vector in semantic space and thus map observed sequences of text data to latent topics. For each document, we assign each context window in a sequence of context windows to a topic. This yields a sequence (or, ignoring order, a distribution) of latent topics that represents the document.

#### The latent variable model.

Consider first a simplified version of the latent variable model (Eq. **1**). The probability of a word *w* being present at some location *t* in the corpus is based on the similarity between its word vector **w** and the latent “gist” at that point in the corpus $c_{t}$, i.e., the discourse vector (15). The word most likely to appear at *t* is the word most similar (closest in semantic space) to the current gist.* The similarities between possible word vectors and the discourse vector can be turned into a probability distribution over words by 1) exponentiating the similarities and 2) dividing by their sum $Z_{c_{t}}$, so that the distribution sums to 1 (Eq. **1**). The gist is latent; $c_{t}$ is a vector in the semantic space that represents the underlying meaning of the current context. Eq. **1** thus associates a distribution over words to every point in semantic space. It also sets up a correspondence between atom vectors (as points in semantic space) and topics. In the generative model (15), the gist makes a slow random walk through semantic space; at each step *t* a word is emitted according to

[1] $$\text{Pr}[w\;\text{emitted}\;\text{at}\;t|c_{t}]=\frac{\exp\;(\langle c_{t},w\rangle )}{Z_{c_{t}}}.$$

This simple model is enough to recover many properties of word embeddings (15).

Arora et al. (17) build on Eq. **1** to give a more realistic generative model. The conditional probability of a word *w* being present at some point *t* in the corpus depends on several factors. It depends on the overall frequency of the word in the corpus, p(w). But it also depends on the local context or “gist” (the familiar $c_{t}$), as well as the global context of the corpus ($c_{0}$). The global context vector $c_{0}$ represents the overall syntactic and semantic structure of the corpus, independent of any local context. The specific combination of local and global context vectors ${\tilde{c}}_{t}$ is a linear combination of $c_{t}$ and $c_{0}$. The relative importance of word frequency and context is controlled by the hyperparameter *α* and local and global context trade-off with hyperparameter *β*. This improved latent variable model is written formally in Eq. **2** and detailed elsewhere (15–18):

[2] $$\text{Pr}[w\;\text{emitted}\;\text{at}\;t|c_{t}]=\alpha p(w)+(1-\alpha )\frac{\exp\;(\langle {\tilde{c}}_{t},w\rangle )}{Z_{\tilde{c_{t}}}},$$

where ${\tilde{c}}_{t}=\beta c_{0}+(1-\beta )c_{t}$ and $c_{0}⊥c_{t}$.

#### Mapping observed words into semantic space.

In the generative direction, Eq. **2** fixes the probability of a word appearing, given details of the corpus and the current gist. In applications, however, we observe the words; the gist is latent. In DATM, we want to infer the gist (i.e., where we are in semantic space) given an observed set of context words and then map this gist to an atom vector. Here we summarize work by Arora et al. (15, 17) that uses this model to derive a theoretically motivated, high-quality embedding of a set of context words: smooth inverse frequency (SIF) embeddings.

Given the generative model in Eq. **2**, we can compute the maximum a posteriori (MAP) estimate of the combined context vector ${\tilde{c}}_{t}$ for a set of context words C (15, 17):

[3] $${\left({\tilde{c}}_{t}\right)}_{\text{MAP}}=\sum_{w\in C}^{}\frac{a}{p(w)+a}w\text{,}\;\text{where}\;a=\frac{1-\alpha }{\alpha Z}.$$

${\left({\tilde{c}}_{t}\right)}_{\text{MAP}}$ is a weighted average of the word vectors in the context window; words are weighted based on their corpus frequency *p*(*w*). Frequent words make a smaller contribution to the estimate of ${\tilde{c}}_{t}$.^†^

For a given set of context words, we now have an estimate of ${\tilde{c}}_{t}$ (recall that ${\tilde{c}}_{t}$ is a linear combination of local gist and global context for the corpus). But we are fundamentally interested in the local gist $c_{t}$. To recover this, we need an estimate of the global context $c_{0}$, which we can then subtract from our estimate of ${\tilde{c}}_{t}$ (17). We first estimate ${\tilde{c}}_{t}$ for a sample of context windows (e.g., sentences) in the data using Eq. **3**. Then we compute the first principal component of the ${\tilde{c}}_{t}$s, recovering the direction with the most variance among the context vectors. We interpret this first principal component as the global context vector $c_{0}$.^‡^ For a given set of context words C, we can estimate $c_{t}$ by using Eq. **3** to compute ${\left({\tilde{c}}_{t}\right)}_{\text{MAP}}$ from the word vectors in C and then subtracting off its projection onto $c_{0}$. The result is an estimate of the latent local gist of C, as a point in semantic space.

Prior work (17, 18) demonstrates that SIF sentence embedding (i.e., weighting word vectors by frequency, summing them, and removing the global context vector) is also an empirically effective representation of the meaning captured by a sentence (or other set of words). In fact, by several metrics, SIF embedding outperforms more sophisticated approaches to represent sentences. Readers familiar with word embeddings may note the correspondence between this MAP and representations of context in the continuous-bag-of-words model (3, 17) (*SI Appendix*).

SIF embedding allows us to map a set of observed words to a location in semantic space. Given that location, we can find the atom vector that is most similar to this estimated gist, i.e., $\arg\;\max_{k\in K}\cos\;\left(k,c_{t}\right)$. This atom vector $k^{\prime }$ then immediately yields the closest topic in semantic space.

We have combined three ingredients—sparse coding of the embedding space (16), the latent variable model (15), and sentence embeddings (17)—into a cohesive procedure that allows researchers to discover latent topics in a corpus and to identify the topic that best matches the estimated gist of an observed context window. Finally, to infer topics across an entire document, we estimate the gist $c_{t}$ over rolling context windows in the document.^§^ This is consistent with a key assumption of the latent variable model: that the gist changes slowly across a document. This last step yields the sequence (or, ignoring order and dividing the topic counts by a normalizing constant, the distribution) of topics underlying the document.^¶^ Here, we code topics as binary variables for each record (present or not).^#^ Taken individually, each component of DATM offers an effective tool for specific tasks and analyses. Once integrated, they generate a strikingly effective and general approach to analyze real-world text data.

## Topics in Descriptions of Violent Death

When we applied DATM to the NVDRS narratives, the resulting 225 topics covered various aspects of violent death. For example, we observed several topics about weapons, substance use, and forensic analyses. To interpret a given topic, we examine the 25 terms closest to the topic’s atom vector and then we assign the topic a label using face validity. We list several topics in Table 1 and all topics in *SI Appendix*.

**Table 1.** Sample topics within narratives of violent death

Topic label	Seven most representative terms
Physical aggression	Tackled, lunged_toward, began_attacking, advanced_toward, attacked, slapped, intervened
Causal language	Sparked, preceded, triggered, precipitated, led, prompted, culminated
Preparation for death	Disposal, deeds, prepaid_funeral, burial, worldly, miscellaneous, pawning
Cleanliness	Unkempt, messy, disorganized, cluttered, dirty, tidy, filthy
Everything seemed fine	Fell_asleep, everything_seemed_fine, seemed_fine, wakes_up, ran_errands, ate_breakfast, watched_television
Suspicion and paranoia	Conspiring_against, plotting_against, restraining_order_filed_against, belittled, please_forgive, making_fun, reminded
Reclusive behavior and chronic illness	Recluse, heavy_drinker, very_ill, chronic_alcoholic, bedridden, reclusive, recovering_alcoholic

Most representative terms are listed in order of highest to lowest cosine similarity to each topic’s atom vector. Topic labels are manually assigned. As part of preprocessing the narratives, we transformed commonly occurring phrases into single terms (29).

**Fig. 1.**
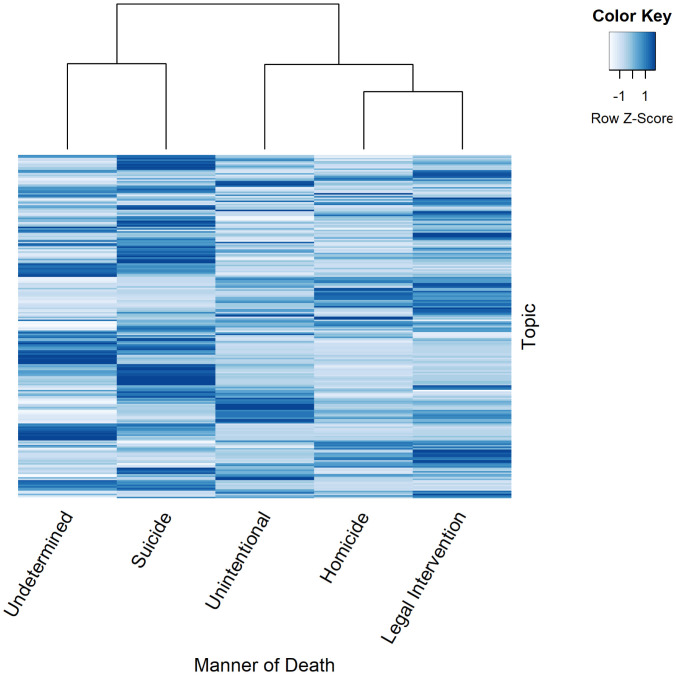
Prevalence of 225 topics in narratives of 272,964 decedents of violent death, by manner of death. Each row represents the fraction of narratives with a given topic by manner of death, with rows standardized across all manners of death.

Fig. 1 illustrates the prevalence of our 225 topics as patterned by manner of death: suicide, unintentional shooting, homicide, homicide resulting from legal interventions (e.g., police shootings), and deaths of undetermined intent. Each row represents the fraction of narratives with a given topic, by manner of death. The dendrogram^‖^ shows that across the manners of death, suicides are most similar in topic distributions to undetermined deaths; this makes sense, because many deaths may look like suicide but lack the required evidence for classification as suicide (22). It also shows that homicides are most similar to legal intervention deaths, reflecting that legal intervention deaths are a unique type of homicide.

### Topics and Latent Semantic Dimensions.

Because the atom vectors corresponding to topics live in an embedding space, we can apply common word-embedding methods to our topics. One prominent deductive approach uses knowledge about cultural connotations to extract a corresponding dimension in the semantic space. Here, we extract a dimension for gender (masculine vs. feminine) in the corpus, following standard word-embedding methods (5).** We then examine the topics that load most highly onto the gender dimension (i.e., have the highest or lowest cosine similarity). Cosine similarity can range from –1 to 1: For gender, the topics with large negative cosine similarity are more distinct to language about men (and not women), while topics with large positive cosine similarity are more distinct to language about women (and not men).

In our data, the most masculine topic is about the military, followed by topics about rural outdoor areas, rifles and shotguns, specific outdoor locations, and characteristics of suspects. The most feminine topic, by contrast, is one about sedative and pain medications, followed by topics about poisoning, children, drug concentrations, and psychiatric medications. Surprisingly, we also observe that a topic about games is highly gendered (i.e., the seventh most masculine topic). This topic reflects a range of games, including video or computer games. Prior work highlights games or forms of play linked to violent death (e.g., Russian roulette, choking games, children playing with guns) (23); the fact that this topic is highly masculine suggests that such deaths may be distinctly patterned by gender.

**Fig. 2.**
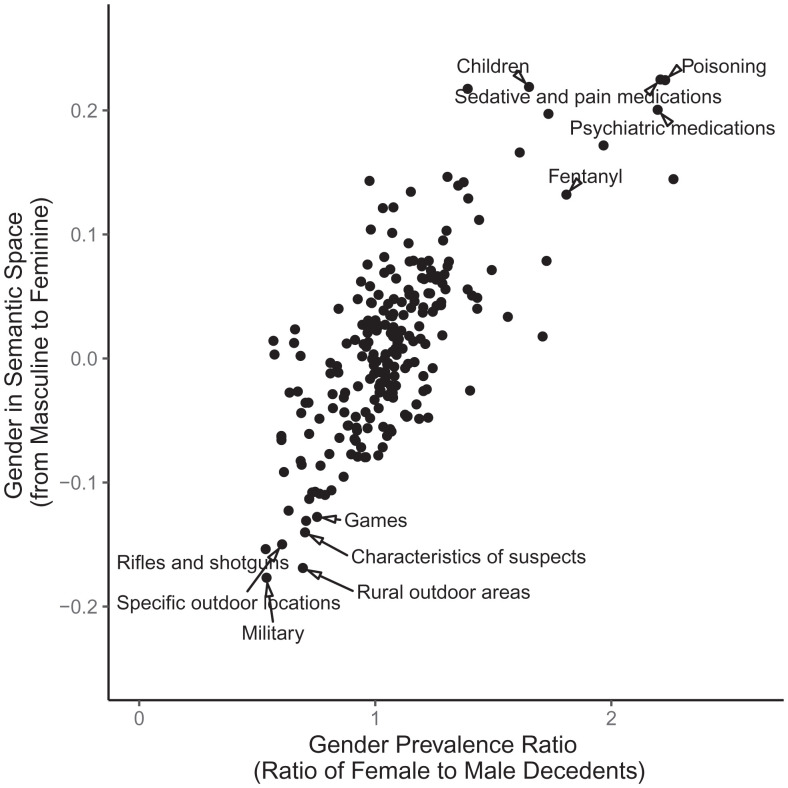
Latent gendered meanings of topics vs. prevalence of topics in female vs. male decedents’ narratives. *N* = 225 topics. For clarity, labels are shown only for topics with high or low gender meanings or gender prevalence ratios; overlapping labels are removed. The *y* axis represents cosine similarity between a given topic and the gender dimension in semantic space. The *x* axis represents the ratio of female decedents’ narratives containing a given topic compared to narratives of male decedents.

In Fig. 2, we compare the cosine similarity of topics to this gender dimension with the mean prevalence of each topic among female victims (versus among male victims). These two variables capture distinct ways that gender is encoded in the NVDRS, which we might expect to be strongly related. Similarity to the gender dimension reflects the appearance of topics in the context of gendered language in the narratives; it can reveal gendered patterns in topics even when there are no corresponding metadata for documents. Mean prevalence captures the extent to which a topic is mentioned among men versus women victims. The strong correlation (Spearman *ρ* = 0.69, *P* < 0.0001) suggests that topics are gendered in semantic space in a way that indeed corresponds to the gender of the victims in the narratives.

Next, we describe two topics in depth. Each one has a high cosine similarity to the gender dimension. We select these topics because they are the most masculine and feminine topics (respectively) that describe weapons of death; weapon use has a well-known gendered pattern in violent death (11, 12). For each topic, we describe the most representative words and the case that loads most highly onto it. Then we use logistic regression to describe correlates of the topic: decedent demographics, manner of death, and number of decedents in the incident, controlling for word count.

#### Topic 141: Rifles and shotguns.

Topic 141 reflects characteristics of long guns (e.g., rifles and shotguns). These firearms are typically owned for hunting and sport shooting (24) and can be used to shoot at far ranges (compared to handguns). The most representative terms refer to makes and models of long guns, as well as characteristics of gun action: how the gun is loaded and fired. The highest-loading case describes the death of a young man accidentally shot by a friend playing with a rifle, who believed it was unloaded. The narrative describes the gun in depth (e.g., as a “bolt action deer rifle”).

Topic 141 is the third most masculine topic in semantic space. This strong gender connotation reflects the fact that violent death by firearm typically involves males (11, 12). Logistic regression confirms that topic 141 is distinctly more common among male victims (than females), controlling for characteristics listed in Table 2 (adjusted odds ratio = 0.49, 95% CI: 0.48 to 0.51). The strong gendered associations of this particular gun-related topic in semantic space (compared to say, topic 61: handguns) could follow from the fact that far more men than women own long guns (24).

**Table 2.** Characteristics of violent deaths with two selected topics

	Topic	
Characteristic	Rifles and shotguns: AOR (95% CI)	Sedative and pain medications: AOR (95% CI)
Female decedent*	0.49 (0.48 to 0.51)	2.52 (2.47 to 2.58)
Decedent race/ethnicity^†^		
American Indian/Alaska Native, NH	1.31 (1.20 to 1.42)	0.46 (0.41 to 0.52)
Asian/Pacific Islander, NH	0.48 (0.43 to 0.54)	0.64 (0.59 to 0.70)
Black or African American, NH	0.88 (0.85 to 0.91)	0.54 (0.51 to 0.56)
Hispanic	0.59 (0.56 to 0.62)	0.63 (0.60 to 0.67)
Two or more races, NH	1.01 (0.92 to 1.10)	0.80 (0.73 to 0.88)
Unknown race, NH	0.70 (0.56 to 0.87)	0.70 (0.56 to 0.87)
Decedent age, y^‡^		
20 to 29	0.96 (0.91 to 1.00)	1.37 (1.29 to 1.46)
30 to 39	0.90 (0.86 to 0.95)	1.74 (1.64 to 1.85)
40 to 49	0.93 (0.88 to 0.98)	1.97 (1.86 to 2.10)
50 to 59	1.03 (0.98 to 1.08)	2.17 (2.04 to 2.30)
60+	1.40 (1.33 to 1.47)	1.68 (1.58 to 1.79)
Manner of death^§^		
Homicide	0.79 (0.77 to 0.82)	0.14 (0.13 to 0.15)
Legal intervention	1.09 (1.01 to 1.17)	0.22 (0.19 to 0.26)
Undetermined	0.06 (0.06 to 0.07)	2.01 (1.95 to 2.07)
Unintentional	3.16 (2.84 to 3.51)	0.13 (0.10 to 0.19)
Multiple decedents in incident^¶^	1.76 (1.68 to 1.84)	0.40 (0.37 to 0.43)
Word count^#^	1.00 (1.00 to 1.00)	1.00 (1.00 to 1.00)

*N* = 272,964 decedents. Topics are coded as present in any amount or not (1/0) in the narrative either of law enforcement reports or of medical examiner/coroner reports. AOR, adjusted odds ratio; NH, non-Hispanic.

*Referent = male.

^†^Referent = non-Hispanic White.

^‡^Referent = 12 to 19.

^§^Referent = suicide.

^¶^Referent = incidents with a single decedent.

^#^The combined word count of both narratives.

We also observe patterns in topic 141 across other covariates. While prior work suggests that the majority of firearm-related decedents are Black (25), our results (Table 2) suggest that patterns may be more nuanced for deaths involving long guns. For instance, this topic is more common among American Indian/Alaska Native decedents and less common in all other race/ethnicity groups (compared to Whites). Finally, the topic was more common in incidents where there were multiple deaths, as one would see in mass shootings. Findings from this topic underscore the need for work on specific guns (26) to more effectively target prevention efforts aimed at firearm control.

#### Topic 53: Sedative and pain medications.

Topic 53 involves sedatives and medications that can be used to control pain. The most representative terms for this topic refer to the names of such medications (e.g., “phenergan”). The highest-loading case describes a middle-aged white male decedent who was found dead next to various prescription bottles with pain medications (e.g., methadone and hydrocodone). The immediate cause of death was ruled as suicide. In general, we found many topics focused on distinct groups of medications and drugs, attesting to the depth and patterned ways in which substances are described in the narratives.

Topic 53 is the most feminine topic in semantic space. This strong feminine connotation may reflect the fact that women are more likely to die by poisoning in suicide (8). Logistic regression confirms that topic 53 is distinctly more common among female victims, controlling for characteristics listed in Table 2 (adjusted odds ratio = 2.52, 95% CI: 2.47 to 2.58). We observe additional patterns of topic prevalence across these correlates. Compared to suicides, this topic is more common in undetermined deaths, but less common in all other deaths. The fact that unclassified deaths disproportionately involve this topic in their narratives resonates with broader scholarship on the misclassification of manner of death. Undetermined deaths are predominately associated with drug intoxication and poisoning (27), and many undetermined deaths involving drugs may be uncounted suicides (28).

These results illustrate that the same methods used to identify the biases or cultural meanings of words in word embeddings can also be used to identify biases of topics extracted with DATM. These methods extend to semantic dimensions beyond gender (4, 14); we provide another example (outdoors versus indoors) in *SI Appendix*.

## Discussion

In this paper, we introduced a method to model topics: DATM. In DATM, topics, sentences, words, and other semantic structures are all represented in a single semantic space. Raw text can be mapped into this space to distill individual documents into sequences of topics and thus draw out the prevalence of topics in a corpus. Using DATM, we discovered a range of themes buried in descriptions of lethal violence from a large administrative health dataset. We observed that the gendering of these topics in semantic space corresponds to the ratio of female versus male victims whose narratives mention these topics and analyzed two highly gendered topics in depth. Methodologically, our model builds on theoretical work to explain word embeddings and represent sentences in embedding spaces (15–17), as well as a wealth of previous models to extract topics (2, 13).

For computational social science and natural language processing, DATM provides an integrated approach to discover patterns in large-scale text data. As a topic model, DATM picks up fine-grained, interpretable topical structures. These topics are coherent despite the fact that no stopwords were prespecified. This makes DATM ideal for real-world applications of text analysis, which are often domain specific and would otherwise require specialized lists of stopwords. Further, DATM offers a cohesive, theoretically motivated approach to integrate questions that are often asked with topic models with questions often asked with embedding methods. A researcher can now ask, for example, not only what topics are in a corpus, but also how these topics load on latent semantic dimensions such as gender or social class.

For public health, our results illustrate patterns encoded in large-scale narrative data about suicides, homicides, and other violent deaths. Using DATM on these data offers a way to break out of the well-worn categorical systems by which we interpret and monitor lethal violence. We found that unstructured text data can hide potential patterns or trends that are not yet part of our standardized menu of structured variables. Such patterns could suggest additional lines of research that aim to reduce violent death, for example, discovering additional indicators of suicide risk, with eventual implications for medical providers or hotlines. Despite the wide use of the NVDRS for research and policy about lethal violence, actionable information in its text data has remained largely out of reach. We hope that DATM will provide an interpretable, flexible, theoretically grounded, and effective tool for scientists to unlock the potential of important datasets like the NVDRS.

## Materials and Methods

Our data are drawn from the NVDRS from 2003 to 2017 (9). This NVDRS database included information about 307,249 violent deaths forwarded from 34 US states and the District of Columbia. This state-level information is abstracted into the NVDRS by public health workers (PHW) using a standardized codebook. We use two text variables in the NVDRS written by PHW: narratives of 1) law enforcement reports and 2) medical examiner or coroner investigative reports. Death records may include one of these variables, both, or none, for a total of 568,262 narratives. We train our word embedding on all of these narratives using word2vec (3, 29). After applying several exclusion criteria, our final sample is 272,964 deaths. For details, see *SI Appendix*.

## Supplementary Material

Supplementary File

## Data Availability

Data cannot be shared. The data used in this study are freely available, but the CDC requires a specific researcher request. Access to the NVDRS data is restricted by the CDC to ensure that individual death data will not be combined with other sources to identify individual cases. Users may apply to the CDC directly for data access: https://www.cdc.gov/violenceprevention/datasources/nvdrs/dataaccess.html. We include information about data access in *SI Appendix* and provide a link to a GitHub project with code: https://github.com/arsena-k/discourse_atoms. Previously published data were used for this work (9).

## Footnotes

*Similarity is measured as the dot product between the two vectors.

^†^The amount of reweighting is controlled by the parameter a. A lower value for a leads to more extensive down-weighting of frequent words, compared to less frequent words. This parameter ranges reasonably from 0.001 to 0.0001; we use a value of 0.001 (17, 18).

^‡^After we subtract off the projection of a specific ${\tilde{c}}_{t}$ onto the global context vector $c_{0}$, the remaining vector $c_{t}$ captures the local context—the gist of what is being talked about. We have removed any information in ${\tilde{c}}_{t}$ that corresponds to what the corpus as a whole usually talks about or how it talks about this (i.e., $c_{t}$ does not just capture frequent words).

^§^Here, we use a context window size with 10 terms.

^¶^Turning the sequence into a distribution accounts for word count differences; it also provides a connection to traditional topic modeling, which associates a distribution over topics to each document.

^#^Note that our approach to assigning topics is fundamentally different from the approach in traditional topic modeling; rather than trying to model each document as a mixture of topics, DATM models each document as a trajectory in semantic space and (in parallel) decomposes semantic space into topical building blocks. The topic closest to each point of the trajectory is then assigned to the document. For this reason, DATM does not take a document-term matrix as input. In a sense, DATM provides a more “bottom–up” approach to inferring topics, rather than a “top–down” approach involving further assumptions about the role of topics in the generative process.

^‖^Computed using hierarchical clustering with Euclidean distance for a similarity metric.

**To extract a gender dimension, we average the vectors for the words woman, women, female, females, she, her, herself, and hers. We then subtract out the average of the vectors for the words man, men, male, males, he, him, himself, and his (5, 14).
