# Supplementary material for: Integrating topic modeling and word embedding to characterize violent deaths
Source: Proc Natl Acad Sci U S A. 2022 Mar 3;119(10):e2108801119. doi: 10.1073/pnas.2108801119 (PMC8915886; doi:10.1073/pnas.2108801119)
Supplement: Supplementary File [file pnas.2108801119.sapp.pdf]

Supplementary Information for

Integrating topic modeling and word embedding to characterize violent deaths

Alina Arseniev-Koehler, Susan D. Cochran, Vickie M. Mays, Kai-Wei Chang, and Jacob Gates Foster

Alina Arseniev-Koehler and Jacob Gates Foster.
E-mails: arsena@g.ucla.edu and foster@soc.ucla.edu.

This PDF file includes:

Figs. S1 to S2
Tables S1 to S4
SI References

Supplementary Information Text

1. Data

As described in the Materials and Methods in the main text, our data are drawn from the National Violent Death Reporting System (NVDRS) collected between 2003-2017. Here we provide additional details on these data. The Centers for Disease Control and Prevention (CDC) share these restricted data with researchers via the execution of a standard use agreement. Users may apply to the CDC directly for data access: https://www.cdc.gov/violenceprevention/ datasources/nvdrs/dataaccess.html.

These data include 307,249 violent deaths, for decedents aged 12 and older: suicides (N=192,115), homicides (N=73,602), deaths of undetermined intent (N=34,266), and 7,266 other deaths such as unintentional deaths (primarily, shootings) and deaths related to legal intervention (e.g., police shootings). Legal intervention deaths are defined using criteria outlined by Barber et. al. (1). As described in the main text, each death record may be accompanied by a narrative of medical examiner/coroner reports, a narrative of law enforcement reports, both narratives, or neither. In total, these data include 302,072 narratives of medical examiner/coroner reports, and 266,190 narratives of law enforcement reports. Initial cleaning of the narratives included corrections for misspelling and minor editing for common abbreviations (e.g., COD: "cause of death"). In the case of multiple death incidents, when the narrative referred to the current victim, "victim" was recoded as "primary_victim" and all other mentions of "victim" were recoded as "extra_victim." We also transformed commonly occurring phrases into single words (i.e., terms) based on collocation (2). The medical examiner/coroner narratives had an average length of 105 terms (SD=77); the law enforcement narratives averaged 120 terms (SD=117). Our resulting corpus from these text variables and pre-processing steps included a vocabulary size of 28,222 unique terms. During training of our embedding (described in SI–Section 2), we removed terms that are in the dataset fewer than 15 times to avoid learning low quality word-vectors for these terms. As described in the main text, we coded topics as binary variables for each death record (present in any amount in either the medical examiner/coroner narrative or the law enforcement narrative = 1, not present in any amount = 0).

We limit our empirical investigation of topic distributions to the 272,979 (88.85%) deaths which have at least 50 terms in either of the narratives. We further exclude 18 deaths where manner of death is missing or is coded as terrorism. This process leaves 272,964 deaths.

In our empirical analyses of the distribution of topics, we used several structured variables in the NVDRS: victim sex (male/female), age at time of death (in years), race/ethnicity, manner of death (suicide, homicide, legal intervention death, undetermined, or unintentional death), and number of victims (1 vs. more than 1). We also used word count of the narrative(s); for cases with narratives of medical examiner/coroner reports as well as narratives of law enforcement reports, word count is combined across both narratives. We coded age into six groups: 12-19, 20-29, 30-39, 40-49, 50-59, and 60 and older. There was no missing data for age or for number of victims. We coded race/ethnicity as: American Indian/Alaska Native, non-Hispanic; Black or African American, non-Hispanic; Hispanic; Two or more races, non-Hispanic; White, non-Hispanic; Asian/Pacific Islander, non-Hispanic; and Unknown race, Non-Hispanic. There was no other missing data in race/ethnicity. To account for missing data for victim sex ($N = 2$), we manually imputed victim sex using information about victim sex described in the narratives (e.g., "victim was a 20 year old male."). See Table S1 for descriptive summaries of these variables.

2. Training the Word Embedding

We trained our word embedding using word2vec with Continuous-Bag-of-Words (CBOW) and negative sampling (3). We did so because of the connections of this architecture to our topic modeling approach (see 4, 5); however, any embedding algorithm can be used to train a semantic space given text data. Using Gensim (2) in Python to train our word embedding, we tuned two hyperparameters: dimensionality of the semantic space and context window size. Specifically, we trained word embeddings with 50, 100, 200, and 300 dimensions, training three models at each dimensionality to vary context window size between 5, 7 and 10, for a total of 12 embeddings. We report context window size as the number of words on each side of the target word. Thus a context window size of 5 means that we use 5 words to the left and 5 words to the right of the target word, for a total of 10 words in the context window C; in general, a context window size n implies a context window C with $2n$ words total. These hyperparameters are within the range of standard choices for hyperparameters (6).

We selected our final word embedding (200-dimensions and a context window size of 5) by comparing the performance of the 12 different embeddings on two common metrics for assessing the quality of embedding models: the WordSim-353 Test and the family section of the Google Analogy Test. The WordSim-353 Test (7) compares the cosine similarity of two words in a word embedding model with similarity assigned by human annotators; our trained word embedding yielded a Spearman correlation of 0.45 ($p<0.0001$) with human-rated similarities. The Google Analogy Test (3) tests how well an embedding model can complete a series of analogies, divided up in various sections (e.g., family, currency, tense, world capitals). We focused on the family section which is most relevant to our data domain. Our trained word embedding correctly completed 70% of the analogies in the family section. We observe that performance on these metrics varied little across our hyperparameters. We set the number of iterations at 10, and negative samples at 5, and we randomly shuffle the order of the documents prior to training our embedding to prevent any ordering effects.

3. Connections between Continuous-Bag-of-Words (CBOW) Word Embeddings and DATM

Word2vec learns a semantic space from a corpus by giving a task to an artificial neural network. In word2vec with CBOW, the task is to guess words from their contexts in the data (i.e., short excerpts of text data, also called context windows). More

precisely, for each context window in the data, a CBOW network (CBOW for short) is asked to predict the most likely word (i.e., target word), given the average of the words in the context window (i.e., the context vector).

This is done across the many possible context windows of data, until CBOW reaches a certain level of accuracy in predicting words. Below, let w_t be the target word (the word at "time", or, equivalently, text position, t), with vector $\mathbf{w}_t$, and and let $c_t = \{w_{t-n}, w_{t-n+1}, \ldots, w_{t-1}, w_{t+1}, w_{t+2}, \ldots, w_{t+n}\}$ be a set of words within a context window C with size n (i.e., $2n$ words total).* Note that we use bold to distinguish vectors from their corresponding entities; so the word-vector $\mathbf{w}_t$ corresponds to the word w_t. Given a set of context words c_t, the probability that CBOW will predict word w_t is given by:

$$P(w_t|c_t) \propto \exp(\langle \mathbf{w}_t, \bar{\mathbf{c}}_t \rangle), \text{ where } \bar{\mathbf{c}}_t = \frac{1}{2n} \sum_{i=t-n, i\neq t}^{t+n} \mathbf{w}_i. \quad [1]$$

CBOW training adjusts the weights so as to maximize the probability of the actual word corresponding to a given context window, for all word/context pairs.

In practice, however, CBOW is trained with two tricks: negative sampling and sub sampling (9). These tricks effectively down-sample more frequently occurring context words (5). Such techniques *implicitly re-weight* the context words, such that a word is guessed from a weighted sum of its context words, where these weights are based on word frequency. This means that in most practical implementations of CBOW (including the one we use), the "context" vector is computed in the same way as context or "gist" in the Discourse Atom Topic Model (c_t), including the down-weighting of frequent context words. Put another way: practical implementations of CBOW learn a semantic space by predicting the most likely word from the estimated "gist"(5), a weighted linear combination of the word vectors. More broadly, this connection implies that CBOW with negative sampling and the Discourse Atom Topic Model actually form a single cohesive theoretical model. In practice, however, any word embedding can serve as input to the Discourse Atom Topic Model.

4. Identifying DATM Topics in a Word Embedding with K-SVD

Here, we describe how the K-SVD algorithm works to identify topics in a trained embedding. As described in the main text, this algorithm outputs a set of K vectors (called "discourse atoms" by 10) such that any of the V word vectors in the vocabulary can be written as a sparse linear combination of these vectors. We refer to these vectors as "atom vectors." As described next, these atom vectors can be interpreted as topics in the embedding space. The words closest to each atom vector typify the topic. K-SVD is a well-established method (11) and we implement K-SVD using the ksvd package in Python (12). Here, we provide details on the K-SVD algorithm to keep the exposition self-contained.†

The input to K-SVD is a matrix $\mathbf{Y}$ of V word-vectors, each of which is N dimensional. Thus, this matrix has N rows and V columns. The goal of applying K-SVD to this matrix is to represent each word-vector as a sparse linear combination of atom vectors, where there are a total of K possible atoms and each is represented by an N dimensional vector. K-SVD output includes two components.

First, the output provides a matrix $\mathbf{D}$ of atom vectors (which we will ultimately interpret as topics), commonly called the dictionary. $\mathbf{D}$ has N rows and K columns; each column is an N-dimensional vector corresponding to an atom in the embedding space. Because these atoms are simply vectors in the same semantic space as word vectors, we can compare them to other vectors (like word-vectors, or latent semantic dimensions) in this space using cosine similarity. To understand what a given atom vector represents, we look at the words in the vocabulary whose word vectors have the highest cosine similarity to each atom vector. Note that, under the Latent Variable Model (described in the main text), these word vectors also give the words most likely to be "emitted" when the context coincides with the atom; this allows us to turn each atom vector into a full-blown topic (i.e., probability distribution over words) as in conventional topic modeling.

Second, the algorithm produces a sparse matrix of coefficients $\mathbf{X}$ with K rows and V columns. Each column in this sparse matrix indicates how a given word can be reconstructed as a linear combination of atom vectors: which atom vectors to use and in what amounts. While we do not use them in this paper, these coefficients could be used to see which words load onto a given topic and with what strengths. Arora et al (10) use these coefficients to disentangle the multiple meanings of words.

Note a key difference between DATM and the more familiar LDA topic modeling: LDA topic modeling decomposes a document-term matrix to find topics; DATM decomposes the embedding matrix. Thus, our approach identifies topics *in the semantic space* of a corpus.

If the output of K-SVD is a good solution, then each word-vector should be well-approximated as a sparse linear combination of atom vectors (i.e., one with few non-zero coefficients). Put another way, using our topics, we should be able to roughly reconstruct the original meanings of the word-vectors. To reconstruct our matrix of word-vectors, we multiply the atom matrix ($\mathbf{D}$) by the coefficient matrix ($\mathbf{X}$). To find a good representation of the original word vectors, we want to minimize the difference between $\mathbf{Y}$ (our word vectors) and $\mathbf{DX}$ (our sparse reconstruction).

Comparing the reconstructed matrix $\mathbf{DX}$ to the original embedding matrix $\mathbf{Y}$ yields measures of error in a discourse atom solution (e.g., sum of squared errors, root mean square error, and even R^2). The approximate decomposition is visualized in Figure S1.

* Note that the implementation of CBOW draws the vector for the context words and the vector for the target word from two different weight matrices. The first vector comes from (averaging) the weights linking the input in CBOW's artificial neural network to the hidden layer. The second vector comes from the weights linking the hidden layer to the output layer. See (8) for a more detailed explanation of the implementation and hyperparameters.

† Throughout we follow the notation and approach of the excellent Wikipedia exposition as well as the original paper (11), fleshing details out and specializing the exposition to our specific case; see: https://en.wikipedia.org/wiki/K-SVD

At the same time, we want a sparse solution; that is, we want to make sure that each word is represented by a small number of topics. Formally, we want to keep the ℓ^0 "norm" of each column in $\mathbf{X}$ (i.e., the number of non-zero elements) small, so that it is less than or equal to the sparsity constraint hyperparameter T_0. Thus, the objective function of the K-SVD constrained optimization problem is:

$$\min_{\mathbf{D},\mathbf{X}} \left\{ \|\mathbf{Y} - \mathbf{DX}\|_F^2 \right\} \tag{2}$$

with the constraint $\|\mathbf{x}_i\|_0 \leq T_0 \quad \forall i$. Recall that $\|\mathbf{x}_i\|_0$ is the ℓ^0 norm of the i-th column of $\mathbf{X}$ and $\left\|...\right\|_F^2$ denotes the Frobenius norm, i.e., the sum of squared entries of the matrix. Hence we want to choose $\mathbf{D}$ and $\mathbf{X}$ such that the total squared difference between the original embedding $\mathbf{Y}$ and the reconstruction $\mathbf{DX}$ is minimized, while constraining each column of $\mathbf{X}$ to T_0 non-zero entries; in other words, a sparse representation of each word vector in terms of the atom vectors.

A. Solving the objective function of K-SVD to arrive at topics. In general, this constrained optimization problem cannot be "solved" (i.e., truly optimized); therefore approximate methods must be used. The overall strategy to minimize the objective function of K-SVD (and thus identify our topics) involves alternating updates to the coefficient matrix $\mathbf{X}$ and the dictionary $\mathbf{D}$. We begin with a randomly initialized dictionary $\mathbf{D}$.

A.1. Updating the coefficients. Given a fixed dictionary, finding the coefficients is basically a least squares problem: we need to find a distinct, sparse linear combination of atom vectors that best represents each word-vector (i.e, each column of the embedding matrix). In K-SVD, this problem is commonly solved (heuristically) with orthogonal matching pursuit (OMP): a greedy algorithm that iteratively finds a sparse representation for each word vector, where the number of atom vectors allowed is determined by T_0 (11, 13). The use of OMP exploits the fact that the minimand $\left\|\mathbf{Y} - \mathbf{DX}\right\|_F^2$ can be rewritten as $\sum_i^N \left\|\mathbf{y}_i - \mathbf{D}\mathbf{x}_i\right\|_2^2$ (note the shift from Frobenius to the familiar ℓ^2 norm). Each of the terms in this sum can be separately minimized with respect to the coefficients $\mathbf{x}_i$ that correspond to the reconstruction of word vector $\mathbf{y}_i$ (with the familiar sparsity constraint T_0 on the number of non-zero coefficients). These separate minimization problems can be addressed using OMP to give an approximate solution (11).[‡]

A.2. Updating the dictionary. Once the coefficients are updated for all columns of $\mathbf{X}'$, we freeze the coefficients. We then update the dictionary of atoms; here we follow (11) closely. We update one atom vector (i.e., column of the dictionary) at a time. To update the kth atom vector, we identify the word vectors whose reconstructions use that atom (i.e., the corresponding coefficient in the sparse representation vector $\mathbf{x}_i$ is nonzero). Now define a representation error matrix $\mathbf{E}_k = \mathbf{Y} - \sum_{j \neq k} \mathbf{d}_j \mathbf{x}_T^j$, where $\mathbf{d}_j$ is the jth column of the dictionary matrix $\mathbf{D}$ (i.e., the atom vector for topic j) and $\mathbf{x}_T^j$ is the jth *row* of the representation matrix $\mathbf{X}$, i.e., all of the coefficients for the jth atom vector. $\mathbf{E}_k$ essentially corresponds to all of the reconstruction error that remains after we have reconstructed $\mathbf{Y}$ with the other $K-1$ topics.

We want to reduce the reconstruction error further by updating the vector for the kth atom $\mathbf{d}_k$ and the corresponding row of the coefficient matrix $\mathbf{x}_T^k$, but we must do so in a way that preserves sparsity. We do so by considering only the columns of the error matrix that correspond to word vectors whose reconstruction currently uses the kth atom, yielding a restricted matrix $\mathbf{E}_k^R$. We likewise restrict $\mathbf{x}_T^k$ to only those elements of the row with non-zero entries (i.e., those coefficients where atom vector k is currently used); call this $\mathbf{x}_R^k$. We now update $\mathbf{d}_k$ and $\mathbf{x}_R^k$ to minimize $\left\|\mathbf{E}_k^R - \mathbf{d}_k \mathbf{x}_R^k\right\|_F^2$; this is, in essence, the "best we can do" to further reduce error by only changing the atom vector $\mathbf{d}_k$ and altering the way that reconstructions *already using* that atom vector load onto it. By construction, this update cannot lead to violation of the sparsity constraint. This sparsity-preserving minimization with respect to $\mathbf{d}_k$ and $\mathbf{x}_R^k$ can be done via singular value decomposition (SVD) of the error matrix $\mathbf{E}_k^R = \mathbf{U}\boldsymbol{\Delta}\mathbf{V}^T$. In essence we want a rank one approximation of the error matrix $\mathbf{E}_k^R$; the optimal such approximation is obtained by setting $\mathbf{d}_k$ to be the first left singular vector (the first column of $\mathbf{U}$) and the reduced coefficient vector $\mathbf{x}_R^k$ to be the transpose of the first right singular vector (the first column of $\mathbf{V}$) times the first singular value (i.e., $\boldsymbol{\Delta}_{11}$). This updating process must be carried out for every column of the dictionary matrix $\mathbf{D}$.

The process iterates between updates to the dictionary and updates to the coefficients (which assign sparse combinations of atoms to each word), until it reaches a predetermined stopping point. In our case, the process stops after either 10 iterations or the total reconstruction error falls below 1×10^{-6}, whichever happens first. The final result is a matrix of atom vectors $\mathbf{D}$ and a matrix of coefficients $\mathbf{X}$ that allow us to reconstruct each vocabulary word as a sparse linear combination of atoms. Conceptually, updating atoms in this way encourages distinct atoms; each time an atom is updated, the goal is to best account for all the variation in words' meanings that the other atoms do not already explain.

We note that our overall approach is extremely *modular*. While we use K-SVD to discretize the semantic space and identify topics, other dictionary learning algorithms (or even clustering algorithms, like k-means) can be used instead.[§] As long as

[‡] See https://en.wikipedia.org/wiki/Matching_pursuit for a simple exposition of the related Matching Pursuit algorithm. OMP works in our case as follows: For a given word vector, we find the closest possible atom vector using cosine similarity. The projection of the word vector onto that first atom vector represents our first attempt at reconstructing the word vector, and hence our first pass at the coefficients. We next compute the residual (the vector difference between the word vector and the reconstruction). We then find the atom vector closest to the residual (i.e., what is not explained by the atom(s) already assigned to this word-vector). This becomes the next atom vector with a non-zero coefficient. In OMP, we compute new coefficients for both atom vectors by projecting the full word-vector onto their span (in this case, a plane); this yields a new set of coefficients and a better reconstruction of the original word-vector. We iterate this process—compute the difference between the word-vector and its current reconstruction; find the atom vector closest to the residual; project the full word-vector onto the span of the iteratively selected atom vectors; repeat—until we have chosen T_0 atom vectors, corresponding to T_0 non-zero coefficients in $\mathbf{x'}_i$ for the sparse coefficient matrix $\mathbf{X}'$ corresponding to the current dictionary $\mathbf{D}'$.

[§] We conducted experiments using k-means. While performance on our corpus was comparable to K-SVD, we found that K-SVD produced interpretable topics more robustly across different corpora. We also note that the "theory of meaning" implicit in the K-SVD approach is more realistic: it views all words as a combination of basic semantic "building blocks" and finds those building blocks with that picture in mind. Using K-means implicitly assumes that the meaning of each word is best represented by the nearest cluster of word vectors, ignoring polysemy.

this discretization returns a set of vectors in the embedding space, those vectors can be interpreted as topics (i.e., probability distributions over words) using the Latent Variable Model. They can also be mapped to the raw corpus using any sentence or document embedding technique to represent a stream of text as a context vector. The modular nature of DATM means that it can be *improved* as an overall strategy for text analysis following any innovation in these components, e.g., improvements to the Latent Variable Model (and SIF embeddings), to dictionary-learning algorithms, or to techniques that map context vectors to atoms.

5. DATM Model Quality and Selecting the Number of Topics

Measuring the quality of a topic model is important to validate that the model is learning human-interpretable topics and to aid in tuning model hyperparameters—most importantly, the number of topics (i.e., atom vectors). Evaluating topic model quality remains an open research area. Given the enormous number of possible models and topics within each model, we employ computational methods to evaluate topic model quality, in addition to human inspection and validation.

We trained candidate K-SVD models with the number of topics/atoms K ranging from 15 to 2000. We then used three metrics to evaluate model quality before selecting our final model. Our three metrics were: coherence, topic diversity (14) and coverage (R^2). Together, these three metrics provide us with interpretable measures for: 1) how internally coherent topics are (coherence); 2) how distinctive topics are from each other (diversity); and 3) how well the topics explain or reconstruct the semantic space itself (coverage). Next, we explain each measure as implemented.

First, coherence is a commonly used family of metrics which attempts to measure the similarity of words within topics in a trained topic model (15–17). To operationalize coherence, we first identified the top 25 word-vectors closest to an atom vector; for each atom vector, we then calculated the average pairwise cosine similarity between these closest word-vectors (17). Finally, we computed the average of these pairwise similarities across all atom vectors to arrive at an overall measure of coherence for the trained model. The coherence metric ranges from 0 to 1, where a value closer to 1 indicates higher average topic coherence (which typically corresponds to human interpretability of the topic, since the corresponding words are semantically similar).[¶] This metric is well suited for topic modeling in embeddings, is efficient to compute, and correlates well with human judgement (17). As illustrated in Figure S2A, we found that models with fewer topics tended to produce slightly more coherent topics, but models were coherent across various numbers of topics.

Second, to measure how distinct topics are, we used an efficient and transparent metric: topic diversity (14, 19). To find diversity, we first identified the 25 word vectors closest to each atom vector in a model (with K atom vectors total). We then computed the proportion of these $25K$ "closest word instances" which are unique to a particular atom. If the top 25 words in every topic are unique, this measure will be 1.0, implying that the topics are very specific and distinct from one another. Topic diversity decreases as a larger number of words are repeated in the top 25 across multiple topics; it would reach its smallest value if the same 25 words were the "top 25" in all topics. As illustrated in Figure S2B, we found that models with fewer topics also tended to produce more distinct topics, and topic diversity dropped rapidly in models with more than approximately 225 topics.

While coherence and diversity favor a parsimonious topic model with few topics, it is nevertheless important that the model "explains" the space of possible meanings in the corpus. To capture this important aspect, we turned to our third metric: coverage. To measure how well the topics in a given model cover the semantic space, we computed the extent to which we could "reconstruct" the original semantic space using just the set of topics. As in k-means—which is in fact a special case of K-SVD (11, 12)—the objective function of K-SVD minimizes the sum of squared errors between the original data and reconstructed data. Using the sum of squared errors and sum of squares total, we computed the proportion of the original variance explained by the topics (i.e., R^2) to measure how well a candidate set of topics explains the semantic space (we refer to the value for R^2 here as coverage). In contrast with topic diversity and coherence, coverage continues to increase in models with more topics, but the marginal gains from adding more topics reduce considerably around 225 topics in our data (Figure S2C).

We selected our final model to balance all three of our metrics for a good quality topic model. Coherence steadily decreased with more topics. Diversity dropped rapidly after around 225 topics. At first, coverage rapidly increased with more topics, but gained little after 225 topics. Thus, we selected a model with 225 topics as our final model. This model had a coherence of 0.59, a diversity of 0.93, and coverage of 0.63 (again, all metrics range from 0 to 1).[‖]

In other applications of K-SVD, Root Mean Square Error (RMSE) or the closely related Sum of Squared Errors (SSE) are used as metrics to select the number of elements (in our case, topics). To further inform our choice of the optimal number of topics, we plotted RMSE (or SSE) against the number of topics, and looked for the point at which adding more topics offers little reduction in SSE or RMSE. Both RMSE and SSE suggest that the optimal number of topics was approximately 250 (Figure S2D), quite close to the value selected by the procedure above balancing coherence, diversity, and coverage.

In Table S2 we list all the topics identified in our data using the Discourse Atom Topic Model. For each topic, we include our label (manually assigned) and the 10 most representative terms (from highest to lowest cosine similarity to the topic's atom vector).

[¶] Hypothetically this coherence metric could range to -1, since cosine similarity between two word vectors in our word embedding may range from -1 to 1. In practice, word-vectors rarely have a negative cosine similarity. For clarity, we report this value as ranging from 0 to 1 in the main text (18).

[‖] The final hyperparameter in the Discourse Atom Topic Model is the sparsity constraint T_0, which is the number of topics that a word in the embedding matrix is allowed to "load" on to (i.e., have a non-zero coefficient). The sparsity constraint must be between 1 (in which case K-SVD is identical to k-means) and the number of topics in the model. We follow Arora et al. (10) in setting the sparsity constraint to 5. As they describe, if this sparsity constraint is not sufficiently low, then some of the coefficients must necessarily be small; this makes the corresponding components indistinguishable from noise (10). We empirically observed that models with more nonzeros have lower coherence and slightly less diversity, but higher coverage.

6. Comparing DATM to Other Topic Models

Topic modeling is a core method in text analysis. It is therefore unsurprising that a plethora of specific approaches exist. These include non-negative matrix factorization, joint-stochastic matrix factorization, matrix rectification, Sparse Additive Generate Models (20), anchor-based topic modeling (19, 21), replicated softmax (22), Latent Semantic Analysis (23) and its variants, and most notably a wide variety of latent Dirichlet allocation (LDA) topic models and implementations (e.g., 2, 24, 25).

DATM differs from this prior work in several ways. Most crucially, DATM differs from the majority of topic models (like LDA) in that it integrates topic modeling with word embedding, capitalizing on the distinct capabilities of each of these core methods. Several other recent topic models also aim to combine word embedding and topic modeling, but do so in ways that are quite different from DATM. Many of these models simply use information about words derived from word embeddings, such as word similarity, to inform the construction of the topic model (e.g., 26–29). In contrast, DATM directly represents a topic in an embedding space (as does 14).

DATM is also distinct from prior topic modeling because it leverages a generative model that connects word embedding *itself* to observed text (i.e., the Latent Variable Model) (4, 5). It does *not* rely on LDA (or any variant of LDA) for a generative model. This is a critical distinction with (14), which represents documents as *mixtures* of topics, as in traditional LDA. DATM, by contrast, represents documents as a sequence of topics, based on a discretization of the inferred context vector position. This sequence can be converted into a distribution over topics, or into a binary presence/absence representation as we do here (or as in 22). As illustrated in this paper, because DATM topics exist directly in the embedding space, researchers can easily extend methods commonly used to work with words in word embeddings (e.g., extracting biases and cultural dimensions, like gender) to work with DATM topics.

Another crucial difference between DATM and other topic models is that its *input* is a semantic space derived from the corpus (i.e., a word embedding trained on the corpus). In contrast, the input to other topic models is usually document-level word counts, e.g., a document-term matrix. To code documents with topics, DATM maps topics onto the sequence of local, inferred context vectors (a "trajectory") that represents each document in semantic space; it thus distills each document into a *sequence* of topics. Ignoring order, this sequence can be converted into a distribution, or even a vector of binary presence/absence indicators. DATM can be thought of as following a "bottom-up" approach to inferring the topics in a document; it is fundamentally different from the traditional, "top-down" approach to topic modeling, which includes further assumptions about the role of topics in the text-generation process.

DATM has several practical advantages compared to many prior topic models. It is robust to stopwords, domain specific vocabulary, and can be used on documents of varying lengths. It can also yield highly interpretable and coherent topics, as we illustrate in this paper. As we show next, the topics identified by DATM are qualitatively different from those picked up with LDA topic models (the mainstream approach in computational social science). We emphasize, however, that the "ideal" topic model for a particular use-case will depend on the researcher's data, theoretical assumptions, and research questions.

A. LDA Topic Modeling on NVDRS Narratives. Here we provide sample topics generated on our data using one of the most popular topic models: LDA topic modeling (Table S3). Our goal is not to show that the Discourse Atom Topic Model necessarily works better than any other topic model *in general*. Rather, our goal is to highlight that our model and LDA pick up qualitatively different topical structures in our data and DATM can answer *different* questions compared to traditional topic modeling approaches.

To train our LDA topic models, we used a Python wrapper (2) for the MALLET implementation of LDA topic modeling (24), after observing that this implementation offered substantially more interpretable topics than the default implementation in Python using the Gensim package (2). For instance, in an LDA model trained with 225 topics using the default implementation, the most five probable terms for one topic (topic 214, selected at random) include: "hispanic," "homeless," "wood," "decomposing," and "inflicted." The five most probable terms for another randomly chosen topic (topic 154) include: "seen_alive," "last," "initiated," "doorway," and "letters." For reference, the overall model had a coherence of 0.11 and a topic diversity of 0.69. As a second example, in an LDA model trained with 100 topics, the most five probable terms for one topic (topic 60, selected at random) include: "garage," "nature," "dog," "fatal_injury," and "contents." This overall model scored similarly on coherence and diversity (0.12 and 0.66, respectively).

We initially tried using the exact same vocabulary as we used for our Discourse Atom Topic Model. However, the resulting topics were uninterpretable. They contained many stopwords and words that are very common in our data and thus lose meaning (e.g., "the" and "victim"). LDA models require careful pre-processing that is specific to the corpus, and often are not robust to stopwords (30–32), unlike the Discourse Atom Topic Model. Thus, for LDA topic modeling, we removed standard stopwords using a list from the nltk package in Python (we retained gender pronouns, however, even though these are considered stopwords in the nltk list). We also removed words that occurred in more than 75% of the documents (ubiquitous words), or fewer than 15 times total in the corpus (very rare words).

To select the best LDA model, we trained 11 LDA models with varying values of K (i.e., topics): 15, 25, 50, 100, 150, 175, 200, 225, 250, 400, and 800. We selected our final LDA topic model among these using coherence and diversity metrics, described in the main paper; coverage does not apply to LDA topic models, since it has to do with the ability of topic atoms to reconstruct an embedding space. To compute coherence and diversity, we selected the "top 25" words for each topic by considering their probability given the topic. In our LDA topic models, coherence has minimal gains after 100 topics (when coherence is 0.18); it then drops with more than 250 topics. Topic diversity begins at 0.66 (at 15 topics, the smallest number of topics we considered) but rapidly diminishes (e.g., by 250 topics the topic diversity is 0.41). Using the elbow method, we

selected a final LDA model with 100 topics to balance both coherence and diversity.

The model with 100 topics has a coherence of 0.18 and topic diversity of 0.49. These metrics suggest that the LDA model captures a more limited and broad (i.e., less coherent and distinctive) set of topics compared to those picked up by the Discourse Atom Topic Model. The fact that these models pick up different kinds of topical structures is illustrated not only by metrics of coherence and diversity, but also by manually inspecting the topics. See all LDA topics in Table S3; this Table includes the topic number and the top 10 most representative words (by probability) for each LDA topic. In comparison with topics picked up by the Discourse Atom Topic Model, these topics tend towards more macro-level themes rather than the nuanced, focused topics identified by the Discourse Atom Topic Model; this is also indicated by the lower average topic coherence. For comparability, we also describe an LDA model with 225 topics (since our Discourse Atom Topic Model used 225 topics). For an LDA model with 225 topics, coherence is 0.18 and diversity is 0.41. We provide 15 randomly selected topics among these 225 topics in Table S4.

LDA topics are not immediately compatible with approaches to identify semantic dimensions in semantic space, e.g., finding a gender dimension, as we do in this paper. However, in datasets (like the NVDRS) that include structured variables (like victim gender) for each document, we *can* examine the prevalence of LDA topics by structured variables. Here, we do so by computing the mean proportion of each topic among female victims, and then doing so again among male victims. Then we divide these two distributions element-wise to identify the topics which are most distinct to women versus men. Note that this is the same procedure we used in the main text to construct the "Gender Prevalence Ratio" in Figure 2.

Using this approach, we find that the LDA topics that are most prevalent in female victims' narratives (versus those of male victims) are LDA topics 90, 77, 9, 70 and 98. Topics 90 and 77 appear to be about interpersonal violence, romantic relationships, and children (for the most representative words, see Table S3). Topics 9 and 70 are about prescription medications, and Topic 98 is about multiple victims. The LDA topics that are most distinctive to male victims' narratives (versus those of female victims) are topics 19, 12, 95, 18, and 78. Topics 19 and 12 are also about romantic relationships and family, but more focused on separation. Topic 95 is about homicides, and topics 18 and 78 are about gunshot wounds and gun actions. Importantly, this approach only captures the *prevalence* of LDA topics by gender. With DATM topics, we can also examine how these topics are gendered in semantic space, i.e., the gendered meaning in their typical language context.

7. Semantic Dimensions Beyond Gender

As we emphasize in the main text, methods used to identify the biases or cultural connotations of *words* can be successfully extended to identify biases of *topics* extracted using DATM. In the main text, we illustrate this important extension of embedding methods using the case of gender. Here, we offer an additional application to illustrate this point: the extent to which topics are associated with descriptions of *indoors* or *outdoors* in the narratives. Indeed, this core approach to measure bias or cultural meaning in topics can be used for *any* strong, stable semantic contrast, including contrasts that may have theoretical motivation but are not (yet) cleanly represented in structured variables.

As with the construct of gender, we extract a dimension for indoors versus outdoors in the corpus. Specifically, we average the vectors for the words: indoors, inside, and indoor, and then subtract out the average of the vectors for the words: outdoors, outside, and outdoor. We examine the topics that load most highly onto the resulting dimension (i.e., have the highest or lowest cosine similarity). Topics with large negative cosine similarity are more distinct to language about outdoors (and not indoors), while topics with large positive cosine similarities are more distinct to language about indoors (and not outdoors). In our data, the most "outdoors" topic is one we had labeled "Specific outdoor locations" (Topic 120), followed by topics labeled "Rural outdoor areas" (Topic 219), and "Canvassed" (Topic 52). The most "indoors" topic is one labeled "Fumes" (Topic 14), followed by topics about "Tubes" (Topic 214), and "Gun Actions" (Topic 152). For context, fumes and tubes both reflect gas poisonings, which occur in closed (i.e., indoor) spaces.

Fig. S1. Decomposing the Embedding Matrix into a Dictionary of Topics and Coefficients

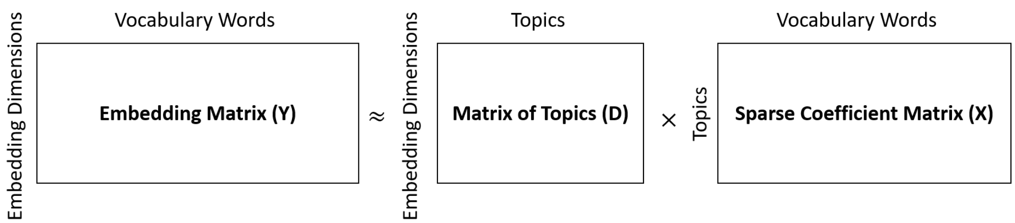

Fig. S2. Measures of Model Quality (Coherence, Diversity, Coverage, and RMSE) against the Number of Topics in the Discourse Atom Topic Model

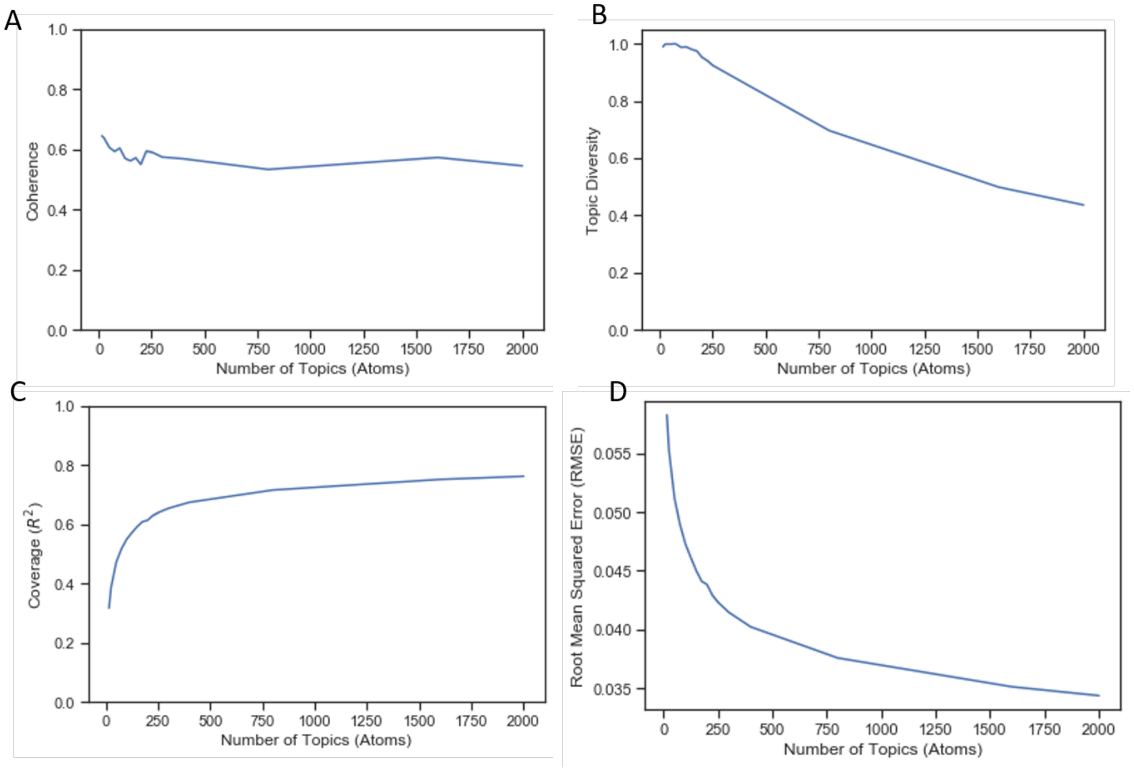

Table S1. Characteristics of Sample of Violent Deaths, drawn from the National Violent Death Reporting System.

Characteristic	N (%) or Mean (SD)
**Female Decedent**[1]	64,404 (23.59%)
**Decedent Race/Ethnicity**	
White, NH	190,474 (69.78%)
American Indian/Alaska Native, NH	4,044 (1.48%)
Asian/Pacific Islander, NH	4,616 (1.69%)
Black or African American, NH	49,218 (18.03%)
Hispanic	19,627 (7.19%)
Two or more races, NH	4,129 (1.51%)
Unknown race, NH	857 (0.032%)
**Decedent Age**	
12-19	18,029 (6.60%)
20-29	61,767 (22.63%)
30-39	49,113 (17.99%)
40-49	52,431 (19.21%)
50-59	46,903 (17.18%)
60+	44,721 (16.38%)
**Manner of Death**	
Suicide	173,006 (63.34%)
Homicide	62,751 (22.99%)
Legal Intervention	5,124 (1.88%)
Undetermined	30,598 (11.21%)
Unintentional	1,485 (0.54%)
**Multiple Decedents in Incident**[2]	13,991 (5.12%)
**Narrative(s) Word Count**[3]	226.37 (158.72)

Notes: N = 272,964. SD= Standard Deviation, NH=Non-Hispanic. [1]Referent = Male. [2]Referent= Incidents with a single decedent. [3]This is the combined word count of the narrative of law enforcement reports and the narrative of medical examiner or coroner investigative report.

Table S2. All 225 Topics identified using the Discourse Atom Topic Model

Topic Number	Topic Label	Top 10 Most Representative Terms
0	Taking (syntactic)	immediate_action, into_protective_custody, easy_way_out, sleeping_pill, antidepressents, pretty_hard, threat_seriously, too_many_pills, bath, own_life
1	Poisoning	mixed_drug_toxicity, ethylene_glycol_toxicity, ethylene_glycol_poisoning, carbon_monoxide_toxicity, acute_combined_drug_toxicity, mixed_drug_intoxication, cyanide_poisoning, combined_drug_toxicity, methadone_intoxication, natural_causes
2	Did not (syntactic)	know_what_happened, make_sense, understand_why, speak_english, hear_anything_else, regain_consciousness, know_why, express_suicidal_thoughts, acknowledge, recognize
3	Ligature around neck	ligature_encircling, encircling, ligature_furrow_encircling, encircles, encircled, fastened_around, tied_tightly_around, partially_encircling, wrapped_tightly_around, ligature_mark_around
4	Head	plastic_bag_covering, plastic_bag_tied_around, bag_covering, butted, blood_pooling_around, blood_pooled_around, fell_backwards_hitting, fell_backwards_striking, shaved, tilted
5	Drug supply	supplier, psychedelic, preferably, induced_psychosis, hallucinogenic, dependencies, sniffed, sells, abusers, users
6	Quotes 1	please_forgive_me, fucking, miss_you, i_hope, i_wish, sic, you_win, ya, whatever_happens, i_guess
7	From (syntactic)	official_sources, aside, borrowed_money, ranging, stealing_items, bad_odor_coming, ranged, refrain, polydrug_toxicity, russia
8	Victim number	1015, 1320, 1445, 1430, 1845, 1345, 1820, 1006, 2334, 1945
9	Knives	folding_pocket, butcher, serrated_edge, serrated_kitchen, switchblade, single_edged, fixed_blade, butterfly, serrated_blade, serrated
10	Specific times	0445, 2330_hours, 1030_hours, 1730_hours, 1445_hours, 1930_hours, 0920, 1330_hours, 0750, 2020_hours
11	Quotes 2	i_hate_my, poor_quality, falling_apart, enjoy_your, generally_unhappy, ruining, ruined, pointless, incompatible, boring
12	Neighborhood locations	fast_food_restaurant, retail_store, restaurant, mall, grocery_store, community_center, convenience_store, supermarket, shopping_center, strip_club
13	Race	american_indian_alaska_native, filipino, american_indian_alaskan_native, obese_caucasian, middle_eastern, indian, overweight_caucasian, asian_pacific_islander, latino, puerto_rican
14	Fumes	fumes, automobile_exhaust, inhaled, propane, natural_gas, gases, inhaling, intentionally_inhaling, exhaust_fumes, hydrogen_sulfide
15	Would (syntactic)	do_whatever, listen, press_charges, press_charges_against, follow, wait_until, join, assure, accept, sue
16	Education	grad, special_education, prestigious, vocational, doing_poorly, technical, rotc, graduate, freshman, junior
17	Around (syntactic)	goofing, noontime, moping, 11_30_pm, staggering, roaming, 7pm, 9_00_pm, doing_chores, started_throwing_things
18	Hedging	most_likely, entirely, definitely, definitively, likely, autoerotic_asphyxiation, natural, represents, strongly, purposeful
19	Up (syntactic)	really_messed, roughed, picks, hooking, sobering, doped, screwing, hose_hooked, messed, flared
20	Stains	stain, brownish, greenish, dark_brown, brown, reddish, dried, pink, blood_soaked, blood_stain
21	Gun shells and cartidges	shell, cartridge, spent_shell, live_round, spent_cartridge, 5_live_rounds, one_live_round, five_live_rounds, spent_round, shotgun_shell
22	Was (syntactic)	still_legally_married, legally_married, very_intelligent, unsure, rather, never_officially_diagnosed, nice_guy, generally_happy, unsure_if, unconfirmed

23	Body posture	wedged_between, hunched_over, resting_against, propped_up, slumped, leaning_against, propped_against, leaning_forward, leaning, slumped_forward
24	Common non-illicit medications 1	docusate, dicyclomine, valsartan, azithromycin, prochlorperazine, fenofibrate, levofloxacin, docusate_sodium, magnesium_oxide, metronidazole
25	Somatic symptoms	dizziness, nausea, headaches, fatigue, diarrhea, discomfort, severe_headaches, chills, stomach_pains, tremors
26	Psychiatric medications	dispensing, antianxiety, anti_seizure, anti_nausea, prescribed_psychotropic, anti_diarrhea, prescritpion, stockpiling, overtaking, mood_stabilizers
27	Multiple (syntactic)	aliases, inpatient_stays, psychiatric_admissions, jurisdictions, personalities, force_injuries, traumas, duis, abdominal_surgeries, knee_surgeries
28	Fall	backwards, cement, cinder_block, backward, stick, concrete, forward, brick, shovel, fell_backward
29	Locations in a house	master_bedroom_closet, bedroom, vicinity, living_room, laundry_room, backyard, sunroom, backroom, master_bedroom, front_yard
30	Clothing	robe, long_sleeve, sweater, boots, gloves, lanyard, drawstring, bathrobe, terry_cloth, rubber
31	Trouble keeping up	hygiene, skills, abilities, diminished, coping_skills, energy, decreasing, stability, cognitive, cognition
32	Not (syntactic)	yet_adjudicated, otherwise_defined, making_sense, otherwise_specified, surgical_candidate, eating_much, feeling_well_lately, mentally_stable, working_properly, strong_enough
33	Physical health conditions in older ages	hernia, diverticulitis, kidney_stones, colostomy, spinal_stenosis, replacements, mrsa, hip_replacement, bilateral_knee, ulcers
34	Grills and gas	charcoal, propane, portable, barbecue_grill, pan, grill, briquettes, burned_charcoal, heater, ash
35	Post-mortem examination	toxicological_examination, toxicological_screen, cat_scan, toxicologic_evaluation, rape_kit, toxicolgy, toxicological_analyses, post_mortem_examination, drug_screen, imaging
36	Clear forensic signs	displayed_obvious_signs, displaying_obvious_signs, certificatedeath_certificate_states, instantaneous, cert, certificate_lists, responding_medics_confirmed, sudden_cardiac, penalty, significant_conditions_contributing
37	Cause of death	hypoxic_ischemic_encephalopathy, anoxic_ischemic_encephalopathy, hypoxic_encephalopathy, hemorrhagic_shock, multisystem_organ_failure, hemothoraces, cerebral_disruption, cardiorespiratory_arrest, hypovolemic_shock, respiratory_arrest
38	Drug user	heroin_abuser, methamphetamine_user, heroin_user, substance_abuser, crack_user, drug_user, meth_user, drug_abuser, crack_cocaine_user, heavily_consume_alcoholic
39	Drug paraphernalia	brown_substance, bottle_cap, plastic_baggie, metal_spoon, wrapper, glass_pipe, cotton_ball, grinder, cut_straw, foil
40	Physical health conditions	hyperlipidemia_hypertension, hyperthyroidism, vitamin_d_deficiency, osteoarthritis, peripheral_vascular_disease, obstructive_sleep_apnea, hypercholesterolemia, bph, peripheral_neuropathy, anemia
41	Miscellaneous observations and evidence	steak_knives, deformed_gray_metal_projectiles, capped_syringes, young_boys, teenage_boys, sealed_envelopes, failed_marriages, abortions, separate_incidents, young_children
42	Approximate time	approximately_30_minutes, approximately_15_minutes, nine_months, 30_mins, 15_mins, eight_months, 20_mins, 15_minutes, thirty_minutes, half_hour
43	Family and domestic	families, chests, wedding_anniversary, mouths, patrol_cars, wives, young_children, cellphones, cell_phones, wallets
44	Drinks	jack_daniels, nyquil, whisky, schnapps, brandy, champagne, bourbon, gin, tequila, rum
45	Physical aggression	tackled, lunged_toward, began_attacking, advanced_toward, attacked, slapped, intervened, shoved, lunged, pepper_sprayed

46	Miscellanous metrics	degrees_fahrenheit, bmi, wh_m, carboxyhemoglobin_saturation, mg_kg, saturation, ut_2014, mg_ml, mcg, co_level
47	Software and devices	gps, software, device, locator, tracking, tracker, heating, monitor, charger, aircraft
48	Paths of weapons into the body	overall_pathway, slightly_upwards, overall_path, temporal_scalp, pathway, downwards, red_purple, parietal_scalp, posteriorly, bullet_pathway
49	Suspicion and paranoia	conspiring_against, plotting_against, restraining_order_filed_against, belittled, please_forgive, making_fun, reminded, reminding, better_off_without, remind
50	Leftover alcohol and drug evidence	empty_liquor_bottles, liquor_bottles, prescription_pill_bottles, pill_bottles, loose_pills, empty_beer_bottles, empty_pill_containers, beer_cans, empty_beer_containers, insulin_syringes
51	Drug-related cognitive disturbances	groggy, lethargic, disoriented, incoherent, agitated, extremely_intoxicated, feeling_better, confused, acting_weird, fine
52	Canvassed	canvassed, nine_9mm_casings, canvased, drug_paraphrenalia, brushy, toured, fleeing, six_9mm_casings, 9mm_shell_casings, canvassing
53	Sedative and pain medications	phenergan, motrin, ultram, flexeril, endocet, lunesta, hydrocodone_apap, skelaxin, amitriptylin, norco
54	Altercation ensued	fight_ensued, gunfire_erupted, physical_altercation_ensued, another_individual, pistol_whipped, gunman, struggle_ensued, scuffle_ensued, suspect, intruders
55	Victim body parts	back_victim30, back_victim9, right_lateral_neck_victim22, back_victim48, chest_victim16, leg_victim37, chest_victim42, back_victim49, back_victim21, graze_type
56	Car crash	totaled, burst_into_flames, t_boned, jacked, totalled, backfiring, wrecking, impounded, intentionally_crashed, wrecked
57	Claims	advised, stated, added, explained, indicated, states, informed, claimed, relayed, said
58	Physical posture	crouching, silhouette, kneeling, northeast, crouched, walkway, platform, stagger, laying, leaning
59	Acute and multi-drug poisoning	acute_diphenhydramine, acute_multidrug, acute_opiate, acute_methanol, acute_salicylate, ethylene_glycol, multidrug, 11_difluoroethane, methanol, salicylate
60	Common non-illicit medications 2	testosterone, coumadin, ativan, antibiotics, estrogen, blood_thinner, dilantin, dilaudid, imodium, norco
61	Handguns	1911_45_caliber, smith_wesson_40_caliber, 380_semi_automatic, hi_point_45_caliber, beretta_9_mm, beretta_9mm, 9mm_hi_point, ruger_22_caliber, ruger_9mm, glock_40_caliber
62	Cleanliness	unkempt, messy, disorganized, cluttered, dirty, tidy, filthy, unclean, untidy, orderly
63	Wounds from physical impact	traumatic_brain, succumbs, superficial_sharp_force, craniofacial, conflagration, eventually_succumbed, massive_facial, facial, sustained_blunt_impact, non_survivable
64	Tried to (syntactic)	conceive, arouse, rouse, reassure, break_free, urinate, dissuade, restrain, assure, establish
65	Cognitive/emotional disturbances and decline	forgetful, irritable, needy, irate, insecure, introverted, moody, aggitated, argumentative, shaky
66	Fentanyl	fentanyl_4_fluoroisobutyryl, narcotic_fentanyl, des_propionyl_fentanyl_intoxication, furanyl_fentanyl_despropionyl_fentanyl, heroin_furanyl_fentanyl, fentanyl_despropionyl_fentanyl, fentanyl_4_fluoro, furanyl_fentanyl, despropionyl_fentanyl, cyclopropyl_fentanyl
67	Toxicology results	cocaine_benzoylecgonine, diazepam_nordiazepam_temazepam, ecgonine_ethyl_ester, nicotine_cotinine, cotinine_nicotine, methadone_eddp, diazepam_nordiazepam, cocaine_cocaethylene_benzoylecgonine, tramadol_o_desmethyltramadol, fluoxetine_norfluoxetine

68	Counseling	counselling, meetings, psychotherapy, outpatient_therapy, alcoholics_anonymous, therapy, counseling, grief_counseling, na_meetings, diversion
69	Cars	cargo_area, idling, drivers_side, windows_rolled_up, lone_occupant, t_boned, rear_hatch, rear_seat, front_seat, hatchback
70	Sums of money	50000, 30000, 20000, 8000, 40000, 100000, 7000, 6000, 3000, 5000
71	Native American	alaskan_native, alaska_native, natural_disease_process, prolonged_substance, soot_stippling, impending_criminal_legal, fabricating, pacific_islander, either, natural_diseases
72	Floor of building	3rd_story, 6th_floor, 2nd_story, 7th_floor, 5th_floor, 4th_floor, 12th_floor, 8th_floor, 10th_floor, 9th_floor
73	Writing materials	folder, notepad, legal_pad, manila_envelope, handwritten_letter, poem, book, poetry, spiral_notebook, folder_containing
74	Body dysfunction	aortic_aneurysm, aneurysm, enlarged_liver, enlarged_heart, abdominal_aortic_aneurysm, ulcer, abscess, umbilical_hernia, ovarian_cyst, enlarged_prostate
75	Psychiatric facilities	psychiatric_facility, psychiatric_ward, psychiatric_unit, psych_ward, mental_health_facility, inpatient_psychiatric_care, psych_unit, involuntarily_hospitalized, behavioral_health_facility, involuntarily
76	Partygoing and substances	partygoers, miscellaneous_items, topics, tested_substances, party_goers, factors, scenarios, ailments, prisoners, circumstanes
77	Chronic disease	atherosclerosis, cholelithiasis, pulmonary_anthracosis, nephrosclerosis, necrosis, fibrosis, aortic_atherosclerosis, hepatic_steatosis, hepatic, left_ventricular_hypertrophy
78	Things to jump or be pushed off	highway_overpass, freeway_overpass, bridge_overpass, railroad_bridge, railroad_trestle, train_trestle, viaduct, walkway, trestle, water_tower
79	Older ages	65, 68, 76, 71, 69, 67, 74, 66, 75, 55
80	Safety	unstated_reasons, safety_reasons, quite_awhile, sake, safe_keeping, actively_looking, safekeeping, mistaken, safety_purposes, quite_sometime
81	Idiopathic health conditions	idiopathic, chronic_fatigue_syndrome, myasthenia_gravis, scleroderma, anorexia_nervosa, diabetes_hypertension_hyperlipidemia, dysthymia, mental_retardation, bipolar_affective_disorder, irritable_bowel_syndrome
82	Long decomposed body	badly_decomposed, partially_skeletonized, frozen_solid, severely_decomposed, charred, partially_decomposed, mummified, heavily_decomposed, dismembered, decaying
83	Death records	examiner_investigator_mei, records_reflect, examiner_opined, investigator_2016_74, professionals, marijuana_card, investigator_2015, investigator_2014, examiners_office, records_show
84	Pawned	jewelry, pawned, fake, pawn, concealed, owning, merchandise, traded, debit_card, valuables
85	Propped open	propped_open, slightly_ajar, slightly_open, cracked_open, ajar, rear_sliding_glass, pried_open, barricading, top_hinge, wide_open
86	Extreme amounts	weighed_230_pounds, weighed_240_pounds, weighed_200_pounds, weighed_250_pounds, 45am, yrs_ago, daughters_ages, 15am, inches_deep, inch_laceration
87	Doing something against one's will	carjack, abduct, dissuade, evade, intubate, persuade, administer_first_aid, reviving, render_first_aid, reconcilliation
88	Prior encounters with the legal system	protective_order, temporary_restraining_order, restraining_order, protection_order, bench_warrant, summons, traffic_ticket, dui_charge, citation, protective_order_against
89	Military guns and weapons	springfield_armory, springfield, hi_point, bersa, walther, highpoint, sturm_ruger, sig_saur, kimber, ruger_lcp
90	Chronic mental instability and substance use	depresion, alcoholism, problematic_alcohol_use, paranoid_schizophrenia, manic_depression, depresssion, chronic_alcoholism, substance_abuse, intravenous_drug_abuse, psychiatric_illness

91	Acting strangely lately	drinking_heavily, drinking_excessively, acting_strangely, exchanging_text_messages, acting_strange_lately, sending_text_messages, acting_paranoid, acting_differently, acting_erratically, acting_strange
92	Unknown details	exact_timeframe, timeline, marital_status, exact_time_frame, if_contributing_condition, timeframe, timeframes, exact_timing, if_contributing_factor, downtime
93	Messages	deleted, unread, unsent, listened, went_unanswered, pinging, sends, forwarded, draft, wireless
94	Tying rope-like materials	tied, fastened, tightened, twisted, tying, wrap, knotted, loosened, looped_around, draped
95	Organ failure	dvt, paroxysmal, venous_insufficiency, elevated_liver_enzymes, esophageal_reflux, pvd, hypertension_atrial_fibrillation, iron_deficiency_anemia, hypo, hcc
96	Hotlines and government institutions	crisis_hotline, crisis_line, law_enforcements, poison_control, police, sheriff_deputies, dispatch, alarm_company, sheriffs_office, law_enforcement
97	As (syntactic)	whow, made_statements_such, categorized, making_statements_such, phrases_such, precaution, wells, best_certified, serves, train_got_closer
98	Over (syntactic)	seas, despondant, despondence, financial_matters, counter_cold_medicine, court_battle, become_increasingly_despondent, counter_sleep_aids, counter_sleeping_pills, hovering
99	Missing, runaway, and endangered	runaway, missing_endangered_person, whow, precaution, teenager, making_statements_such, made_statements_such, categorized, missing_endangered, dispute_words_exchanged
100	Out of doors	landscape, plumbing, fish, roofing, heating, construction, catering, ski, sports, fields
101	Case number 1	1174, 1170, 328, 907, 949, 491, 617, 766, 486, 708
102	Games	games, game, video_game, volleyball, computer_games, dominoes, beer_pong, football_game, tennis, chess
103	Everything seemed fine	fell_asleep, everything_seemed_fine, seemed_fine, wakes_up, ran_errands, ate_breakfast, watched_television, woke_up, ate_dinner, woke
104	Blood alcohol level	096, 179, 218, 0_08, 247, 0_02, 0_01, 246, g_100_ml, 390
105	News and official reports	news_reports, news_articles, court_records, press_release, da_press_release, newspaper_reports, court_documents, da, media_reports, newspaper_articles
106	First aid and CPR	administered_cpr, fire_dept, initiated_cpr, first_aid, continued_cpr_until, began_cpr_until, rescue_personnel, basic_life_support, initiated_cpr_until, swat
107	Lacerations	lacs, fresh_cuts, elbows, superficial_incisions, thighs, slicing, punctures, slash_marks, shins, cut_marks
108	Transfer to medical institution	life_flighted, trasnported, air_lifted, medflighted, readmitted, transfered, upon_admission, despite_resuscitative_measures, mental_ward, airlifted
109	Involved parties	involved_party2, involved_party3, involved_party1, related_party1, concerned_party2, witness3, witness4, reporting_party, related_party2, involved_party
110	Young adults	youths, young_men, juveniles, individuals, teenagers, women, men, students, adults, parties
111	Characteristics of suspects	uid, tank_top, hoody, hooded_sweatshirt, tar_heroin, dreads, velvet, sheep, tarry, ski_mask
112	Intentions and desires	intending, willing, must, wanted, wants, suppose, didn_t_want, intended, pretending, wanting
113	Quotes 3	happiness, i_truly, sic, hate, i_wish, life_sucks, i_hope, god_bless, i_hate, soul
114	Quotes 4	loves, hated, hates, loved, will_miss, proud, worthless, felt_like, hoped, disappointment

115	Employer-related	apparant, excellent__employee, occasional__drinker, illegal__immigrant, abandoned__warehouse, illegal__alien, unnamed__citizen, avid__hunter, off__duty__firefighter, addictive__personality
116	Body parts	lower__lung__lobe, ulna, upper__lung__lobe, subclavian, psoas__muscle, humerus, 10th__rib, hemidiaphragm, subclavian__artery, axilla
117	Gangs and criminal networks	gang, rival__gang, bloods, crips, gang__activity, drug__trade, crips__gang, rival, rival__gang__members, revenge
118	Large amounts	delayed__effects, lots, hundreds, bunch, large__number, variety, excessive__amounts, substantial__amount, large__quantity, large__amount
119	Absence of signs and information	circumstantial__info, circumstancial__info, narrative__available, foul__play__suspected, detectable__pulse, ages__given, success, fixed__address, brain__activity, elaboration
120	Specific outdoor locations	drainage__ditch, ravine, grassy__area, pasture, vacant__lot, wooded__area, pond, field, public__park, natural__area
121	Chairs	lounge__chair, folding__chair, recliner__chair, plastic__lawn__chair, lawn__chair, rocking__chair, reclining__chair, cross__legged, porch__swing, chair
122	Went to do something	their__separate__ways, grocery__shopping, lie__down, golfing, buy__cigarettes, bowling, bike__ride, lay__down, do__laundry, cool__off
123	Filth and disarray	walls, human__feces, blood__smears, smears, stacked, scattered__throughout, clutter, broken__glass, bloody__footprints, dirty__dishes
124	Projectile	metal__projectile, jacketed__bullet, jacketed__projectile, lead__bullet, gray__metal__projectile, copper__colored__projectile, jacket__fragment, copper__jacket, copper__jacketed__bullet, copper__jacketed__projectile
125	On (syntactic)	weekly__basis, may__X__20XX, numerous__occasions, many__occasions, depending, regular__basis, daily__basis, private__property, occassion, operating__table
126	Housekeeping	housekeeping, maid, desk__clerk, cleaning__staff, housekeeping__staff, housekeeper, hotel__staff, maintenance__staff, motel__staff, hotel__management
127	Recent social interactions	visited, texted, emailed, text__messaged, spoken, passed__away, confided, sent__text__messages, expressed__suicidal__ideations, split__up
128	Mass murder	children__ages, unspent__rounds, daughters__ages, blocks__away, inch__barrel, 380__caliber__casings, wheelers, wheeling, deformed__gray__metal__projectiles, 40__caliber__cartridge__casings
129	Harassing	harassed, stalked, harrassed, unfaithful, consoled, taken__advantage, disrespectful, remodeled, held__hostage, victimized
130	Case number 2	2205, 2111, 0002, 1526, 2038, 1113, 1853, 2039, 1719, 2134
131	Out of air	smothering, oxygen__displacement, helium__gas__inhalation, helium__inhalation, oxygen__exclusion, oxygen__deprivation, neck__compression, upper__airway__obstruction, vitiated__atmosphere, oxygen__depletion
132	Medical professionals	neurologist, mental__health__provider, psychologist, specialist, mental__health__professional, counselor, psychotherapist, therapist, psychiatrist, pain__management__doctor
133	Discoloration (graphic)	discoloration, purple, purge, bluish, fly__eggs, discolored, bright__red, coloring, blistering, lips
134	Rifling through car	rifled, coursing, sunroof, rear__passenger__window, passenger__side__window, rear__window, sliding__glass__door, windshield, window__blinds, backdoor
135	Enforcement agency	kentucky, highly__decomposed, southern, bureau, bordering, highway__patrol, file__prince__george, new__york, council, corrections
136	Problematic arrest	apparant, illegal__immigrant, extensive__criminal__record, ak__47__rifle, abandoned__warehouse, excellent__employee, occasional__drinker, exacto__knife, upcoming__court__appearance, extention__cord
137	Finances	bonds, funds, credit, 6000, 401k, checkbook, stocks, 8000, 20000, bank__card
138	Acting strangely, drug-related	act__strange, freaking__out, poking, act__strangely, seizing, snore, convulsing, physically__fighting, harass, fussing

139	Died in hospital	extubated, intubated, asystolic, never_regained_consciousness, declared_brain_dead, condition_declined, rhythm, asystole, surgical_intensive_care_unit, pulseless_electrical_activity
140	Turmoil	turmoil, discord, strife, friction, difficulties, insecurity, instability, interpersonal, disagreements, romantic
141	Rifles and shotguns	savage_arms, bolt_action, savage, pump_action, mossberg, stoeger, stevens, 410, remington, 12_gauge_winchester
142	Things to hang from	ceiling_rafter, ceiling_rafters, metal_beam, stair_railing, spiral_staircase, ceiling_beam, light_fixture, ceiling_joist, support_beam, bedpost
143	Alcohol	malt_liquor, 24_oz, budweiser, bud_light, 40oz, 40_ounce, 24oz, 16_oz, miniature, miller_lite
144	Military	reserves, air_force, us_army, army, national_guard, armed_forces, afghanistan, active_duty, navy, tour
145	Sitting at	sofa, dining_room_table, dining_table, kitchen_table, park_bench, couch, carpeted_floor, kitchen_counter, television_stand, weekly_basis
146	Proof	proven, ascertained, fingerprinted, proved, established, reviewed, deemed, processed, swabbed, photographed
147	Uncertainty	unlikely, uncertain_whether, too_late, looks_like, unclear_why, seems, speculated, looked_like, rumored, raining
148	Escalation	becoming_increasingly, becoming_more, become_more, increasingly, noticeably, notably, become_increasingly, profoundly, grown_increasingly, generally
149	Illegal narcotics	crystal_meth, crystal_methamphetamine, illegal_narcotics, spice, recreational_drugs, synthetic_marijuana, meth, crack_cocaine, heroin_e, herion
150	Mental illnesses	ocd, obsessive_compulsive_disorder, bipolar_disorders, borderline_personality_disorder, generalized_anxiety_disorder, mania, schizoaffective_disorder, oppositional_defiant_disorder, delusional_disorder, borderline_personality
151	Amounts of substances	045, 050, 040, 060, 026, 025, cocaine_cocaethylene_benzoylecgonine, 018, 083, 047
152	Gun actions	always_carried, cleaning_equipment, enthusiast, dry_firing, battle_ensued, cleaning_kit, 357_cal, malfunctioned, reloaded, accidentally_discharged
153	Institutional involvement	mandated, sponsored, technical, administrative, mandatory, representative, educational, assistant, vocational, restoration
154	Limited information and evidence	supplemental, supplementary, redacted, very_limited, concludes, conflicting, contains_little_useful, extremely_limited, gives, extremely_brief
155	Brain trauma	hemorrhages, basilar_skull_fractures, cerebral_contusions, ecchymoses, hemorrhaging, subscalpular, bilateral_periorbital, subgaleal_hemorrhage, subdural_hemorrhages, fractures
156	Incarceration	cell_block, bunk, top_bunk, segregated, cell, segregation_unit, maximum_security, solitary_confinement, correctional_institute, guards
157	Letters	multi_page, computer_generated, farewell, hand_written, titled, typed, entitled, outlining, sticky, detailing
158	States and countries	california, florida, arizona, colorado, mexico, texas, united_states, oklahoma, minnesota, ny
159	Observers	passers, passer, drowning_complicated, attendant_doctor, ingesting_pills, pass_surgery, complicated, intoxication_complicated, telemetry, county_coroner
160	Drinking	consumed_large_quantities, occasionally_drank, consumed_large_amounts, consuming_large_amounts, smoked_cigarettes_drank, consumes, smelled_strongly, consuming_large_quantities, smelled_heavily, consume_large_amounts
161	Past suicidal attempts and ideation	commited, contemplated, commiting, commits, x74, comitted, contemplating, would_often_threaten, contemplate, committ
162	Recluse behavior and illness	recluse, heavy_drinker, very_ill, chronic_alcoholic, bedridden, reclusive, recovering_alcoholic, forgetful, mentally_unstable, legally_blind

163	Surveillance	surveillance_camera, surveillance_video, security_camera, video_surveillance, footage, surveillance_footage, security_cameras, convience, surveillance_cameras, security_footage
164	Cognitive actions	deliberation, awaking, pleading_guilty, reviewing, learning, shorty, thorough_investigation, noticing, gaining_access, gaining_entrance
165	Appointments	twenty, follow_up_appointment, scheduled_appointment, appoint, appointment_scheduled, seventeen, thirty, forty, appointment, surgical_procedure
166	Numbers	six, seven, eight, four, five, 6, twelve, 00_buck, 4, nine
167	Dead body position	face_down, facedown, fetal_position, non_responsive, supine, hunched_over, lifeless, fully_clothed, nude, sitting_upright
168	Self-injurious behavior	starving, joked_about_killing, isolating, suspending, may_have_harmed, asphyxiating, defended, intentionally_suffocating, voluntarily_committed, suspend
169	Older and middle-aged life stressors	digestive, gender_identity, economic, experiencing_intimate_partner, urinary_tract, having_martial, circulatory, pending_legal, impulse_control, gastro_intestinal
170	Police-related	reponded, tracked_down, evading, kansas_state, headquarters, arresting, baltimore_city, canvassed, eluding, uniformed
171	Sneaky movements	snuck, darted, blacking, neatly_laid, lashed, lashing, spaced, hangs, nodding, chickened
172	Extra victim	extra_victim3_extra, extra_victim5, 453, 1173, 492, 447, 808, 301, 749, 988
173	Seemed like	apprehensive, fifteen_minutes_later, seemed_excited, nobody_cares, very_secretive, ten_minutes_later, 150_feet, nobody_cared, half_hour_later, items_strewn
174	Digital communication	snapchat, facebook_message, tm, snap_chat, tms, message, emails, text, text_message, disturbing_text_message
175	Metrics	qty, g_respectively, bmi, wh_m, 06, mcg, 90_remaining, merged_into_ky_2017, olds, mg_kg
176	People	guy, girl, young_girl, liar, boy, young_man, kid, woman, gangster, mutual_friend
177	Forensic analyses	studies, analysis, antemortem, analyses, ante_mortem, inconclusive, non_contributory, prolonged_hospitalization, laboratory, postmortem_toxicological
178	Waning engagement	dozed, dozing, tapered, brushed, label_torn, taper, grid, fend, cooled, tapering
179	With (syntactic)	having_difficulty_dealing, having_trouble_dealing, medics_pronouncing, coupled, dealt, certainty, obsessed, strained_relationships, interfering, coincided
180	Suicidal	self_destructive, suidical, overt, sucidal, subtle, passive, suicidial, suicial, self_harm, fatalistic
181	Living situation	retirement_community, transitional_housing, boarding_house, rooming_house, senior_living_facility, low_income, transitional, halfway_house, transient_lifestyle, independent_living
182	Preparation for death	disposal, deeds, prepaid_funeral, burial, worldly, miscellaneous, pawning, distributed, giving_away, pre_paid
183	Right side of body	right_temporal_bone, proximal, right_frontal, frontal_bone, inferior, right_temporal, basilar_skull, overlying, parietal_scalp, skull_base
184	Descriptions of time sequence	shortly_after, after, shortly_thereafter, upon, eventually, shortly_before, subsequently, af, upon_arrival, moments_later
185	Personality and behavior	weird, grumpy, cheerful, loopy, moody, irritable, shy, upbeat, quiet, pleasant
186	In (syntactic)	addition, 1994, 1970, 1995, 2017_1078, late_afternoon_hours, meantime, 2002, 1999, 1997
187	Month	april, september, june, february, october, august, november, march, july, january

188	Observations	apparant, abandoned_warehouse, occasional_drinker, excellent_employee, illegal_immigrant, empty_wine_glass, unsent_text_message, addictive_personality, unnamed_citizen, extensive_criminal_record
189	Making (syntactic)	amends, bad_decisions, inappropriate_comments, similar_comments, advances_towards, advances_toward, bad_choices, poor_decisions, similar_statements, statments
190	Relationships	strained_relationships, interacted, having_sexual_relations, sexual_relationship, flirting, certainty, sexual_relations, beef, interacting, conversing
191	Forced entry	single_wide_mobile, split_level, forcibly_entered, invaded, burglarized, trashed, ranch_style, complete_disarray, condemned, quarrel_location
192	Rope materials	nylon_strap, bungee_cord, tow_strap, nylon_belt, nylon_cord, ratchet_strap, yellow_nylon_rope, shoestring, tow_rope, necktie
193	Belongings	belongings, permission, pension, loan, social_security_benefits, possessions, stuff, groceries, gifts, food_stamps
194	Decomposed body (graphic)	bloated, bloating, mummification, maggots, skin_slippage, marbling, insect_activity, blistering, maggot_activity, partially_mummified
195	Loud noises	loud_crash, loud_thump, pop_noise, loud_boom, loud_thud, loud_sound, boom, popping_sound, loud_pop_sound, muffled_bang
196	Scheduled event	doctors_appointment, court_hearing, court_appearance, meal, counseling_session, christmas, holiday, thanksgiving, follow_up_appointment, valentine
197	Children	biological, eldest, newborn, grandchild, foster, youngest, toddler, fathered, molesting, sexually_abusing
198	Frequency of time 2	may_14th_2015, numerous_occasions, many_occasions, weekly_basis, several_occasions, operating_table, 23rd_2015, depending, regular_basis, 11th_2015
199	Hostile interactions	home_invasion_robbery, card_game, drug_transaction, gunfight, shootout, scuffle, hostage_situation, verbal_exchange, confrontation, brawl
200	Substance dependency	nicotine_dependence, generalized_anxiety, narcotic_dependence, recurrent_major, opiate_dependence, episodic, hypothyroid, alcholism, bulemia, recurring
201	Illegals	illegal_immigrant, ongoing_issue, abandoned_warehouse, absentia_case, unnamed_citizen, armed_security_guard, illegal_alien, active_restraining_order, extensive_criminal_record, outstanding_felony_warrant
202	Decay	whose_skeletal_remains, spouses, only_ones, alcoholics, heroin_addicts, recovering_alcoholics, heavy_drinkers, manners, strangers, though_its_contents
203	Crimes	grand_larceny, felony_menacing, criminal_mischief, misdemeanor, simple_assault, criminal_trespass, reckless_endangerment, felony_theft, assault_battery, retail_theft
204	Justification	ruled_justifiable, remains_unsolved, 558, gang_motivated, pedestrian_vs_train, road_rage, random_violence, justifiable_self_defense, 3289, considered_justifiable
205	Traffic	tractor_trailer, semi_tractor_trailer, semi_truck, into_oncoming_traffic, dump_truck, westbound, eastbound, swerving, median, southbound
206	Treatment	resistant, began_administering, intensive, ect, life_saving, receives, currently_undergoing, requiring, undergoing_radiation, discontinued
207	Interpersonal violence	extra_victimuspect, prime, pn, coh, physical_altercation_ensued_between, began_attacking, claiming_self_defense, person5, gh, hep
208	Water	shore_line, boat_ramp, waterway, waters, fresh_water, dam, basin, partially_submerged, harbor, downstream
209	Cancers	lymph_nodes, malignant, lymph_node, metastases, lesion, adenocarcinoma, cancerous, pancreas, metastatic, metastasized

210	Financial problems	having_financial_difficulties, having_financial_problems, experiencing_financial_problems, experiencing_financial_difficulties, financial_problems, struggling_financially, financial_difficulties, having_financial_troubles, financial_issues, bankruptcy
211	Short-range weapons (knives and handguns)	steak_knife, 380_caliber_handgun, kitchen_knife, 40_caliber_handgun, butcher_knife, 38_caliber_handgun, 45_caliber_handgun, 9mm_handgun, 32_caliber_revolver, 357_caliber_handgun
212	Expressed suicidal ideations	expressed_suicidal_ideations, voiced_suicidal_ideations, knee_surgery, nervous_breakdown, expressed_suicidal_ideation, expressed_suicidal_thoughts, pacemaker_installed, miscarriage, hip_surgery, verbalized_suicidal_ideations
213	Drug concentrations	concentrations, levels, concentration, toxic_levels, toxic, lethal_levels, therapeutic_levels, elevated_levels, toxic_level, production
214	Tubes	tubing_connected, tube_attached, tubing_leading, tube_connected, tubing, tubing_attached, plastic_tubing, helium_gas_tank, nitrogen_tank, plastic_tube
215	Sexual body appendages	penis, right_arm, arm, right_leg, genitals, throat, breasts, right_ankle, fingers, inner_thigh
216	Worker	contractor, worker, co_worker, coworker, landscaper, construction_worker, customer, employee, maintenance_worker, delivery_person
217	Causal language	sparked, preceded, triggered, precipitated, led, prompted, culminated, may_have_contributed, occured, completely_unexpected
218	Containers	suitcase, garbage_can, cardboard_box, dresser_drawer, plastic_container, beer_can, briefcase, drawer, bible, pill_bottle
219	Rural outdoor areas	wooded, densely, mountain, farmer, rural, hiker, forested, muddy, swamp, picnic_area
220	Body fluids	splatters, oozing, urine_samples, saturated, coagulated, congealed, exuded, large_puddle, smeared, thinning
221	Foul odor	foul_odor, foul_smell, bad_odor, bad_smell, foul_odor_coming, mail_piling_up, strong_foul_odor, foul_order, foul_smell_coming, strong_odor_coming
222	Semi-auto pistol manufacturers	springfield_armory, springfield_arms, davis_industries, bersa, hi_point, springfield, walther, kimber, sig_sauer, cobra
223	Directions	west, south, east, north, south_side, west_bound, westbound, northbound, avenue, southbound
224	Family members	mother, grandmother, father, sister, niece, aunt, stepfather, brother, fiance, best_friend
Most representative terms are listed in order of highest to lowest cosine similarity to the topics vector. Misspellings which were not caught in preprocessing are retained here. Topics which are observed to be syntactic are denoted with (syntatic) in the topic label. Topics which may be graphic are denoted with (graphic) in the topic label. One term is modified in this table to "may_X_20XX" to retain anonymity.		

Table S3. All 100 Topics identified using LDA Topic Modeling

Topic Number	Top 10 Most Representative Terms
0	stating, phone, text_message, received, text, i_m, stated, left, message, text_messages
1	bedroom, bed, floor, lying, deceased, living_room, kitchen, room, discovered, face_down
2	report, death, nothing_further, police, alive, pronounced, medical, unresponsive, incident, manner
3	heroin, drug, cocaine, history, drugs, unresponsive, drug_abuse, methamphetamine, drug_paraphernalia, marijuana
4	hanging, neck, rope, suicide, ligature, closet, cut, belt, tree, basement
5	suicide, death, history, note, report, medical, law_enforcement, manner, called_911, medications
6	depressed, recently, job, lost, due, suicide, depression, family, problems, recent
7	night, morning, bed, hours, evening, sleep, sleeping, couch, called_911, woke_up
8	blood, observed, located, noted, mouth, body, appeared, feet, top, side
9	prescription, pills, overdose, oxycodone, empty, medication, bottle, medications, bottles, filled

10	ago, months, 3, years, 5, 4, 2, weeks, 6, days
11	death, states, police, pronounced, scene, method, medical, 58, white, alcohol
12	girlfriend, roommate, exgirlfriend, relationship, told, broken__up, night, recently, roommates, kill__himself
13	medical, emergency, service, scene, pronounced, arrived, declared, 1814, plethora, ev
14	details, died, unspecified, time, firearm, place, residence, body, age, location
15	suspect, homicide, murder, killed, charged, arrested, fled, altercation, kill, killing
16	gun, shot, head, pulled, put, trigger, firearm, guns, shooting, fired
17	mention, attempts, note, diagnosis, depressed__mood, mental__health, information, intentional, given__regarding, threats
18	gunshot, shot, weapon, wounds, chest, recovered, handgun, fired, shooting, multiple
19	wife, divorce, separated, estranged, told, children, problems, kill__himself, marriage, spouse
20	stated, witness1, time, told, spoke, witness2, left, located, back, mentioned
21	pain, back, suffered, chronic, surgery, medications, due, doctor, years, chronic__pain
22	suspect1, suspect2, suspects, hispanic, shot, robbery, murder, charged, arrested, suspect3
23	death, unresponsive, undetermined, manner, history, pronounced, intoxication, drug, signs, methadone
24	residence, white, back, house, 46, 54, yard, backyard, 60, died
25	hospital, transported, died, admitted, injury, local, days, staff, complications, transferred
26	reported, incident, reports, died, family, time, occurred, decedent, believed, date
27	black, unknown, shot, circumstances, 19, age, relationship, 18, suspects, age__race
28	wound, head, gunshot, self__inflicted, revolver, handgun, weapon, 38__caliber, hand, 22__caliber
29	cancer, white, diagnosed, health, care, recently, dementia, 69, doctor, 72
30	work, day, morning, show__up, co__worker, employer, worked, failed, working, called
31	suicide, attempted, depression, history, attempts, attempt, past, previous, overdose, suicidal__ideations
32	father, parents, school, family, 16, 17, 15, 14, 18, grandfather
33	unresponsive, responded, scene, alive, report, pronounced, death, medical, physical, reported
34	door, room, locked, open, hotel, entered, opened, motel, checked, check
35	store, parking__lot, business, owner, back, building, restaurant, office, local, employee
36	house, fire, inside, body, set, home, trailer, church, due, property
37	information, report, apparent, regarding__circumstances, nature, suicidal, dead, toxicology, positive, suffering
38	multiple, neck, chest, knife, stabbed, stab__wounds, injuries, stab, times, abdomen
39	apartment, neighbor, neighbors, heard, window, floor, apartment__complex, door, resident, inside
40	dead, reportedly, subject, white, body, confirmed, suicidal, scene, recently, death
41	alcohol, abuse, history, problem, 44, drugs, 36, 37, scene, previous
42	death, information, time, ruled, police, toxicology, report, additional, responded, determined
43	brother, sister, family, law, family__members, home, family__member, nephew, day, house
44	money, state, pay, move, living, years, financial__problems, rent, property, bills
45	told, asked, wanted, back, leave, needed, thought, talk, started, refused
46	white, death, due, 28, 35, gunshot, wound, 56, female, homocide
47	home, returned, left, day, earlier, find, return, work, morning, returning
48	son, white, home, 57, died, 62, 63, residence, 64, 65
49	officer, officers, police, fired, attempted, shot, times, began, involved, stop
50	white, died, 45, 48, 47, 53, 43, 38, 42, residence
51	residence, foul__play, deceased, circumstance, investigators, adult, firearm, evidence, signs, approximately
52	depression, medication, taking, history, anxiety, medications, prescribed, suffered, doctor, diagnosed
53	made, suicide, past, threats, suicidal, depressed, kill__himself, told, wanted, talked
54	argument, arguing, began, drinking, leave, threatened, started, left, argued, house
55	medical, history, diabetes, hypertension, disease, pressure, due, suffered, high__blood, significant
56	days, welfare__check, check, contact, requested, residence, deceased, called, unable, landlord
57	vehicle, car, driver, parked, truck, driving, drove, seat, road, side
58	deceased, white, residence, 30, scene, medical, history, 40, 31, suffered
59	wound, head, gunshot, handgun, pistol, weapon, semi__automatic, gun, firearm, exit
60	witness, bar, people, party, involved, witnesses, fight, group, altercation, street
61	scene, death, discovered, recovered, observed, responded, unknown, pronounced, related, manner
62	medical, emergency, service, scene, pronounced, responded, unresponsive, circumstanced, experiencing, unmentioned
63	note, suicide, notes, left, written, letter, life, addressed, family, stating
64	history, mental__health, disorder, depression, bipolar__disorder, treatment, diagnosed, schizophrenia, facility, bi__polar

65	emergency, hospital, transported, medical, room, service, pronounced, arrival, department, shortly__after
66	died, 22, 20, white, 24, 23, 21, incident, report, case
67	residence, white, 50, 51, 59, inside, scene, complainant, incident, discovered
68	information, incident, provided, noted, 25, 26, cousin, unknown, residence, died
69	prior, incident, day, months, weeks, month, week, days, approximately, released
70	medications, prescription, prescribed, clonazepam, depression, included, gabapentin, trazodone, citalopram, alprazolam
71	circumstances, prior, unknown, white, history, home, source, mental__health, treatment, clear
72	officers, related__party, hours, noted, medical, located, advised, examiner, stated, responded
73	shot, gun, wound, head, died, african__american, own__life, indicate__why, homocide, killed
74	death, manner, medical, examiner, 49, 41, coroner, ruled, dead, arrived
75	left, wound, back, death, entrance, brain, exit, bullet, upper, front
76	suicide, head, white, basement, bag, plastic__bag, note, death, discovered, secured
77	female, boyfriend, children, child, 39, relationship, exboyfriend, woman, custody, pregnant
78	gunshot, wound, head, self__inflicted, rifle, 22__caliber, heard, intra__oral, single, chin
79	missing, body, reported, area, park, wooded__area, located, woods, water, river
80	alcohol, drinking, fiance, beer, alcoholic, bottle, intoxicated, abuse, alcoholism, drank
81	law__enforcement, arrived, called, stated, called__911, call, advised, deceased, 911, laying
82	died, circumstances, result, manner, day, prior, death, responded, notes, services
83	friend, friends, house, told, night, day, staying, called, asked, time
84	mother, home, told, grandmother, day, uncle, mom, stepfather, aunt, earlier
85	police, called, arrived, told, call, responded, received, find, 911, department
86	prior, days, years, 52, 55, approximately, months, past, due, weeks
87	wound, chest, shotgun, self__inflicted, gunshot, head, weapon, 12__gauge, single, legs
88	bathroom, blood, floor, left, bathtub, wrist, cut, wrists, noted, shower
89	death, manner, toxicology, positive, blood, ethanol, autopsy, arrived, talked, showed
90	female, husband, white, home, exhusband, kill__herself, problems, history, estranged, attempted
91	scene, dead, pronounced, called, white, 27, 32, 29, 33, 34
92	suicide, note, left, exwife, depressed, committed, commit, stating, depression, contents
93	2, 1, 3, deceased, due, 4, residence, information, time, hispanic
94	jail, arrested, prison, charges, released, court, arrest, cell, case, probation
95	multiple, black, wounds, homicide, gunshot, suspect, street, suffering, motive, information
96	garage, suicide, car, inside, carbon__monoxide, poisoning, running, white, note, vehicle
97	daughter, heard, called, phone, told, called__911, house, home, arrived, upstairs
98	victim1, victim2, extra__victim2, victims, merged__into, victim3, female, shot, wounds, killed
99	injuries, head, blunt__force, trauma, jumped, struck, multiple, bridge, hit, train
Most representative terms are listed in order of highest to lowest probability of being generated by each LDA topic. Misspellings which were not caught in preprocessing are retained here.	

Table S4. Random Sample of 15 Topics identified using LDA Topic Modeling with 225 Topics

**Topic Number**	**Top 10 Most Representative Terms**
38	room, hotel, motel, hotel__room, checked, motel__room, manager, staying, day, bed
44	witness, heard, witnesses, gunshots, stated, ground, street, area, scene, ran
87	white, died, 67, 68, residence, suicide, home, 75, grandson, 80,
94	police, white, park, death, report, 65, passerby, scene, state, deceased
97	shot, gun, head, wound, self__inflicted, shoot__himself, self__inflcited, tested__negative, himslef
107	stated, spoke, wanted, thought, knew, past, lot, aware, mentioned, hurt
111	job, lost, depressed, recently, due, losing, work, quit, loss, unemployed
123	argument, arguing, fiance, argued, night, gotten__into, drinking, left, leave, began
124	told, wanted, called, phone, talking, talked, talk, day, die, spoke
149	heroin, unresponsive, history, drug, cocaine, intoxication, drug__paraphernalia, fentanyl, syringe, morphine
152	incident, prior, day, 47, night, days, fatal, date, took__place, informed
163	3, 2, 4, 5, months, years, 6, hours, prior, approximately
166	details, unspecified, died, firearm, time, place, residence, body, age, location
185	hospital, transported, died, local, admitted, injuries, ambulance, expired, transferred, surgery

191	medical, emergency, service, scene, pronounced, notified, considered, clear_whether, moments_before, pronounces
Most representative terms are listed in order of highest to lowest probability of being generated by each LDA topic. Misspellings which were not caught in preprocessing are retained here.	

References

1. C Barber, et al., Homicides by police: comparing counts from the national violent death reporting system, vital statistics, and supplementary homicide reports. *Am. journal public health* **106**, 922–927 (2016).
2. R Řehůřek, P Sojka, Software Framework for Topic Modelling with Large Corpora in *Proceedings of the LREC 2010 Workshop on New Challenges for NLP Frameworks.* (ELRA, Valletta, Malta), pp. 45–50 (2010).
3. T Mikolov, K Chen, G Corrado, J Dean, Efficient estimation of word representations in vector space in *arXiv preprint arXiv:1301.3781.* (2013).
4. S Arora, Y Li, Y Liang, T Ma, A Risteski, A latent variable model approach to pmi-based word embeddings. *Transactions Assoc. for Comput. Linguist.* **4**, 385–399 (2016).
5. S Arora, Y Liang, T Ma, A simple but tough-to-beat baseline for sentence embeddings in *5th International Conference on Learning Representations, ICLR 2017.* pp. 1–16 (2016).
6. J Pennington, R Socher, C Manning, Glove: Global vectors for word representation in *Proceedings of the 2014 conference on empirical methods in natural language processing (EMNLP).* pp. 1532–1543 (2014).
7. Y Matias, et al., Placing search in context: the concept revisited. *ACM Transactions on Inf. Syst.* **20**, 116–131 (2002).
8. X Rong, Word2vec parameter learning explained in *arXiv preprint arXiv:1411.2738.* pp. 1–21 (2014).
9. T Mikolov, Wt Yih, G Zweig, Linguistic regularities in continuous space word representations in *Proceedings of the 2013 Conference of the North American Chapter of the Association for Computational Linguistics: Human Language Technologies.* (Association for Computational Linguistics, Atlanta, Georgia), pp. 746–751 (2013).
10. S Arora, Y Li, Y Liang, T Ma, A Risteski, Linear algebraic structure of word senses, with applications to polysemy. *Transactions Assoc. for Comput. Linguist.* **6**, 483–495 (2018).
11. M Aharon, M Elad, A Bruckstein, K-svd: An algorithm for designing overcomplete dictionaries for sparse representation. *IEEE Transactions on signal processing* **54**, 4311–4322 (2006).
12. R Rubinstein, M Zibulevsky, M Elad, Efficient implementation of the k-svd algorithm using batch orthogonal matching pursuit, (Computer Science Department, Technion), Technical report (2008).
13. YC Pati, R Rezaiifar, PS Krishnaprasad, Orthogonal matching pursuit: Recursive function approximation with applications to wavelet decomposition in *Proceedings of 27th Asilomar conference on signals, systems and computers.* (IEEE), pp. 40–44 (1993).
14. AB Dieng, FJ Ruiz, DM Blei, Topic modeling in embedding spaces. *Transactions Assoc. for Comput. Linguist.* **8**, 439–453 (2020).
15. M Röder, A Both, A Hinneburg, Exploring the space of topic coherence measures in *Proceedings of the eighth ACM international conference on Web search and data mining.* pp. 399–408 (2015).
16. D O'callaghan, D Greene, J Carthy, P Cunningham, An analysis of the coherence of descriptors in topic modeling. *Expert. Syst. with Appl.* **42**, 5645–5657 (2015).
17. N Aletras, M Stevenson, Evaluating topic coherence using distributional semantics in *Proceedings of the 10th International Conference on Computational Semantics (IWCS 2013)–Long Papers.* pp. 13–22 (2013).
18. J Mu, P Viswanath, All-but-the-top: Simple and effective post-processing for word representations in *6th International Conference on Learning Representations, ICLR 2018.* pp. 1–25 (2018).
19. S Arora, et al., A practical algorithm for topic modeling with provable guarantees in *International Conference on Machine Learning.* pp. 280–288 (2013).
20. J Eisenstein, A Ahmed, EP Xing, Sparse additive generative models of text in *Proceedings of the 28th international conference on machine learning (ICML-11).* (Citeseer), pp. 1041–1048 (2011).
21. S Arora, R Ge, A Moitra, Learning topic models–going beyond svd in *2012 IEEE 53rd annual symposium on foundations of computer science.* (IEEE), pp. 1–10 (2012).
22. GE Hinton, RR Salakhutdinov, Replicated softmax: an undirected topic model. *Adv. neural information processing systems* **22**, 1607–1614 (2009).
23. S Deerwester, ST Dumais, GW Furnas, TK Landauer, R Harshman, Indexing by latent semantic analysis. *J. Am. society for information science* **41**, 391–407 (1990).
24. AK McCallum, Mallet: A machine learning for language toolkit. http://mallet.cs.umass.edu (2002).
25. DM Blei, AY Ng, MI Jordan, Latent dirichlet allocation. *J. machine Learn. research* **3**, 993–1022 (2003).
26. J Petterson, et al., Word features for latent dirichlet allocation. in *NIPS.* pp. 1921–1929 (2010).
27. H Zhao, L Du, W Buntine, A word embeddings informed focused topic model in *Asian Conference on Machine Learning.* pp. 423–438 (2017).
28. P Xie, D Yang, E Xing, Incorporating word correlation knowledge into topic modeling in *Proceedings of the 2015 conference of the north American chapter of the association for computational linguistics: human language technologies.* pp. 725–734 (2015).

29. R Das, M Zaheer, C Dyer, Gaussian lda for topic models with word embeddings in *Proceedings of the 53rd Annual Meeting of the Association for Computational Linguistics and the 7th International Joint Conference on Natural Language Processing (Volume 1: Long Papers)*. pp. 795–804 (2015).
30. J Boyd-Graber, D Mimno, D Newman, Care and feeding of topic models: Problems, diagnostics, and improvements. *Handb. mixed membership models their applications* **225255** (2014).
31. A Schofield, M Magnusson, D Mimno, Pulling out the stops: Rethinking stopword removal for topic models in *Proceedings of the 15th Conference of the European Chapter of the Association for Computational Linguistics: Volume 2, Short Papers*. pp. 432–436 (2017).
32. A Schofield, M Magnusson, D Mimno, Understanding text pre-processing for latent dirichlet allocation in *Proceedings of the 15th conference of the European chapter of the Association for Computational Linguistics*. Vol. 2, pp. 432–436 (2017).
